# Global transcriptional analysis of nitrogen fixation and ammonium repression in root-associated *Pseudomonas stutzeri *A1501

**DOI:** 10.1186/1471-2164-11-11

**Published:** 2010-01-07

**Authors:** Yongliang Yan, Shuzhen Ping, Junping Peng, Yunlei Han, Liang Li, Jian Yang, Yuetan Dou, Yan Li, Huili Fan, Ying Fan, Danhua Li, Yuhua Zhan, Ming Chen, Wei Lu, Wei Zhang, Qi Cheng, Qi Jin, Min Lin

**Affiliations:** 1 Biotechnology Research Institute, Chinese Academy of Agricultural Sciences, Key Laboratory of Crop Biotechnology, Ministry of Agriculture, Beijing 100081, China; 2 State Key Laboratory for Molecular Virology and Genetic Engineering, Institute of Pathogen Biology, Chinese Academy of Medical Sciences, Beijing 100176, China; 3 National Centre for Plant Gene Research, Beijing 100101, China; 4 TwistDX Ltd., Babraham Research Campus, Cambridge CB22 3AT, UK

## Abstract

**Background:**

Biological nitrogen fixation is highly controlled at the transcriptional level by regulatory networks that respond to the availability of fixed nitrogen. In many diazotrophs, addition of excess ammonium in the growth medium results in immediate repression of *nif *gene transcription. Although the regulatory cascades that control the transcription of the *nif *genes in proteobacteria have been well investigated, there are limited data on the kinetics of ammonium-dependent repression of nitrogen fixation.

**Results:**

Here we report a global transcriptional profiling analysis of nitrogen fixation and ammonium repression in *Pseudomonas stutzeri *A1501, a root-associated and nitrogen-fixing bacterium. A total of 166 genes, including those coding for the global nitrogen regulation (Ntr) and Nif-specific regulatory proteins, were upregulated under nitrogen fixation conditions but rapidly downregulated as early as 10 min after ammonium shock. Among these nitrogen fixation-inducible genes, 95 have orthologs in each of *Azoarcus *sp. BH72 and *Azotobacter vinelandii *AvoP. In particular, a 49-kb expression island containing *nif *and other associated genes was markedly downregulated by ammonium shock. Further functional characterization of *pnfA*, a new NifA-σ^54^-dependent gene chromosomally linked to *nifHDK*, is reported. This gene encodes a protein product with an amino acid sequence similar to that of five hypothetical proteins found only in diazotrophic strains. No noticeable differences in the transcription of *nifHDK *were detected between the wild type strain and *pnfA *mutant. However, the mutant strain exhibited a significant decrease in nitrogenase activity under microaerobic conditions and lost its ability to use nitrate as a terminal electron acceptor for the support of nitrogen fixation under anaerobic conditions.

**Conclusions:**

Based on our results, we conclude that transcriptional regulation of *nif *gene expression in A1501 is mediated by the *nif-*specific and *ntr *gene regulatory systems. Furthermore, microarray and mutational analyses revealed that many genes of unknown function may play some essential roles in controlling the expression or activity of nitrogenase. The findings presented here establish the foundation for further studies on the physiological function of nitrogen fixation-inducible genes.

## Background

Biological nitrogen fixation, a process utilized only by certain prokaryotes, is catalyzed by a two-component nitrogenase complex [1]. Nitrogen-fixing microorganisms colonize a wide variety of habitats and can be found free-living in soils and water, in association with grasses, or in root-nodule symbioses with legumes. Consequently, they have evolved sophisticated regulatory networks that respond to multiple environmental cues [2]. Fixed nitrogen, such as ammonium, is one of the most important environmental signals that regulate nitrogen fixation. Regulation of *nif *gene expression has been most extensively studied in diazotrophic proteobacteria [3]. It is becoming increasingly apparent that the genes necessary for nitrogen fixation in many diazotrophs have common structures and functions. However, the mechanisms by which cellular nitrogen levels are sensed and nitrogen signals are transmitted can vary considerably among different nitrogen-fixing bacteria [4].

The availability of diazotrophic genome data can provide insights into the gene regulatory network of nitrogen-fixing bacteria. Whole-genome microarray platforms are appropriate for the study on the environmental responses and the global regulation of diazotrophs. For example, this approach was used to analyze the genes that regulate nitrogen fixation and Fe (III) reduction in *Geobacter sulfurreducens *[5]; analyze the general stress responses of *A. vinelandii *during the carbon and nitrogen diauxic shift [6]; perform transcriptome analysis of *Sinorhizobium meliloti *during symbiosis [7]; identify expression islands clustered on the symbiosis island of the *Mesorhizobium loti *genome [8]; and perform transcriptomic studies of *Bradyrhizobium japonicum *in the bacteroid state [9] and during chemoautotrophic growth [10]. In addition, Rediers *et al*. [11] demonstrated the successful application of a *dap*-based *in vivo *expression technology selection strategy to identify *P. stutzeri *A1501 genes that are switched on during rice root colonization and off during free-living growth. An analysis of NifA-dependent transcription patterns using a competitive hybridization method led to the identification of three novel NifA-regulated genes (*nrgA*, *nrgB*, and *nrgC*) in the symbiotic region of *B. japonicum *[12]. Collectively, these data suggest that a large fraction of the proteins potentially involved in the control of nitrogen fixation remain uncharacterized. We believe that a more complete characterization of these genes will shed light on the underlying molecular mechanisms of nitrogen fixation and contribute to our understanding of the evolution of nitrogen-fixing bacteria.

A1501 is a versatile soil bacterium that exhibits the unusual feature, for a *Pseudomonas*, of being capable of nitrogen fixation, and it is capable of endophytic association with rice plants [11,13-19]. This strain has received particular attention because of its specific metabolic properties, which include denitrification under anaerobic conditions, nitrification under aerobic conditions, and nitrogen fixation under microaerophilic conditions [14]. The association of A1501 with crop plants is a promising model system for the study of plant-microbe-soil interactions in the rhizosphere. The response of bacteria to fixed nitrogen has often been studied after short-term exposure to excess ammonium concentrations, which we refer to as ammonium shock. Although the genetic and physiological responses to fixed nitrogen concentrations have been well studied in A1501 and other diazotrophic strains, the response of nitrogen-fixing bacteria to ammonium shock has not yet been examined using DNA microarray analysis. In an effort to better understand the transcriptional regulation of *nif *gene expression, we took advantage of the availability of the complete genome sequence of A1501 to construct a genomic array system that we used to profile the global transcription of A1501 under nitrogen fixation and ammonium shock conditions. We identified 255 upregulated genes under nitrogen fixation conditions, 166 of which were found to be collectively downregulated as early as 10 min after ammonium shock. Among these nitrogen fixation-inducible genes, 95 have orthologs in *Azoarcus *sp. BH72 and *A. vinelandii *AvoP. Finally, we identified a new gene, termed *pnfA*, which might play a role in the control of nitrogenase activity.

## Results

### Overview of expression profiling analysis

We previously investigated the global gene expression profile of A1501 under nitrogen fixation and nitrogen excess conditions and identified a total of 549 genes that exhibited more than a two-fold change in expression under nitrogen fixation conditions [20]. Among these genes, the expression of 255 genes was dramatically enhanced and the expression of 294 genes was strongly repressed (see Additional file 1). The upregulated genes mainly belonged to three different functional categories: nitrogen metabolism-related genes, including a set of nitrogen fixation-related genes; a large number of genes encoding transporter and membrane proteins; and genes with unknown function. Note that 46 hypothetical genes, covering 20% of all the up-regulated genes, were upregulated under nitrogen fixation conditions; however, the physiological significance of these genes is unknown.

To identify genes that are differentially expressed in response to changes in nitrogen availability, we used a whole-genome DNA microarray and real-time RT-PCR to analyze global transcription under nitrogen fixation and nitrogen-excess conditions. The expression of 429 genes was significantly altered under ammonium shock conditions compared to nitrogen fixation conditions, including 186 genes whose expression was downregulated and 243 genes whose expression was upregulated. These alterations in gene expression are summarized in Additional file 2. A large percentage of upregulated genes were associated with growth rate and included those involved in information storage and processing (e.g., DNA replication, recombination, and repair; transcription and translation; and ribosomal structure and biogenesis). In addition, genes associated with energy production/conversion and nutrition-related metabolism (e.g., amino acid, nucleotide, lipid, and coenzyme metabolism) were also significantly upregulated. Short exposure of the bacteria to excess ammonia induced the expression of various genes for translation apparatus, including forty-four genes encoding many ribosomal proteins, three genes (*rpoA*, *rpoB*, and *rpoZ*) encoding the DNA-directed RNA polymerase, and five genes encoding protein translation factors (*infAB*, *tsf*, *tuf*, *efp*, and *greA*) and the ribosome-binding factor (*rbfA*). The rapid increase in expression of these genes was first observed after 10 min of ammonium shock, whereas the levels of expression of most other known genes responsible for growth and metabolism were not significantly changed. Thus, our results indicate that upon short-term exposure of A1501 to excess ammonium, the increase in the expression of ribosomal protein-associated genes precedes the increase in the expression of most genes required for growth.

### Identification of genes induced specifically under nitrogen fixation conditions

**Figure 1:**
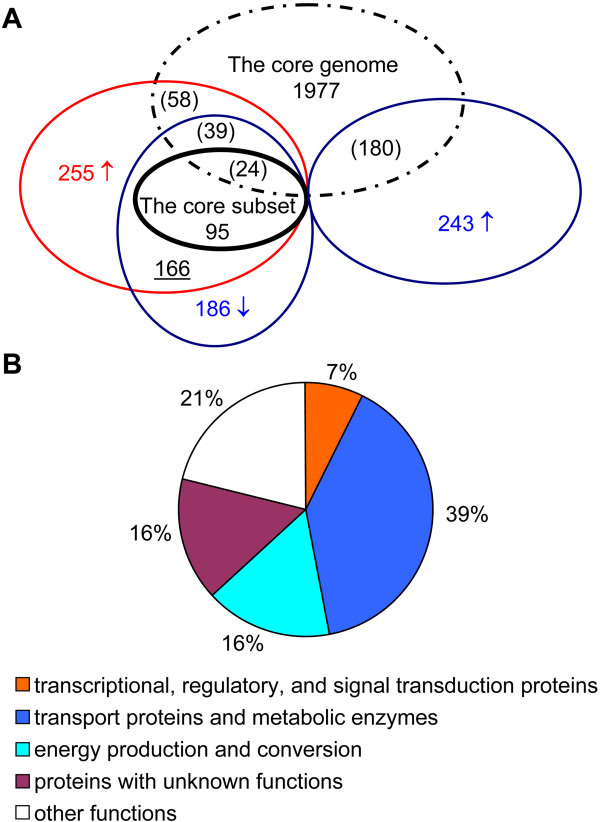
**Overview of expression profiling analysis**. (A) Venn diagram showing the number of genes differentially regulated under nitrogen fixation (red arrow) and ammonium shock conditions (blue arrow). The bold circle indicates the core subset of nitrogen fixation-inducible genes. The dotted circle indicates the *Pseudomonas *core genome. The numbers of upregulated and downregulated genes found in the *Pseudomonas *core genome are shown in parentheses. The number of genes induced specifically under nitrogen fixation conditions is underlined. (B) Functional categories of the core subset of nitrogen fixation, condition-induced genes.

An analysis of the phylogenetic relationship between A1501 and other diazotrophic strains based on comparison of 16S rRNA gene sequences revealed that A1501 is most closely related to *A. vinelandii *AvoP and *Azoarcus *sp. BH72. This finding is consistent with results from the phylogenetic analysis based on NifH proteins of diazotrophic strains (data not shown). In this study, we found that 166 genes were upregulated under nitrogen fixation conditions but were rapidly downregulated as early as 10 min after ammonium shock, indicating a regulon of nitrogen fixation-inducible genes in A1501 (for a detailed description, see Additional file 3). Genomic sequence data for the three most closely related diazotrophic bacterial species, A1501, *A. vinelandii *AvoP, and *Azoarcus *sp. BH72, are publicly available [20-22]. Thus, studies of the A1501 regulon genes shared by the three related diazotrophic strains allowed us to delineate a core subset of nitrogen fixation-inducible genes. Among these genes identified in A1501, 95 have orthologs in *Azoarcus *sp. BH72 and *A. vinelandii *AvoP. We therefore designated these 95 genes as part of the core subset of the regulon induced specifically under nitrogen fixation conditions (Figure 1A). The core subset can be classified into several functional categories, and the relative occurrence of genes belonging to each category is shown in Figure 1B. In addition to the *nif *genes (20%) which are directly involved in the synthesis, maturation, and function of nitrogenase, there are other core genes that encode four major functional groups of proteins, including transcriptional, regulatory, and signal transduction proteins (7%); transport proteins and metabolic enzymes (39%); proteins involved in energy production and conversion (16%); and proteins of unknown function (16%). Note that a very high proportion of the proteins involved in energy metabolism are induced under nitrogen fixation conditions, which is in accordance with the well-known fact that biological nitrogen fixation is a highly energy-dependent process, requiring large amounts of both reducing power and ATP. The detailed implications of the changes in protein expression levels will be determined in the future. When more genomic sequence data of other root-associated diazotrophs becomes available, the current list of 95 genes in the core subset will further decrease.

### Transcriptional analysis of the nitrogen fixation island in A1501

**Figure 2:**
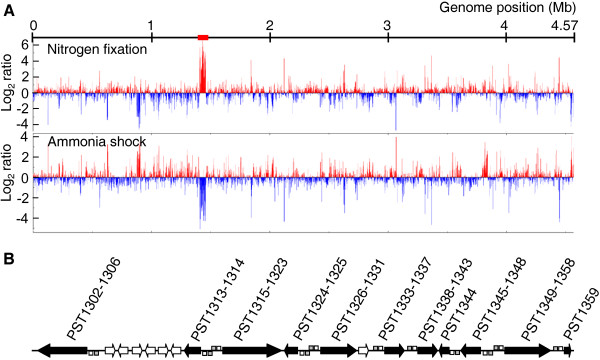
**Identification of an expression island in the A1501 genome that supports nitrogen fixation**. (A) Schematic representation of the regions in the A1501 chromosome with upregulated and downregulated gene expression. Upregulated (red lines) and downregulated (blue lines) genes under nitrogen fixation and ammonium shock conditions. The 49-kb *nif *gene expression island (red bar) is located in the chromosome (from PST1302 to PST1359). (B) Predicted operons (black arrows) with conserved RpoN and NifA promoters. The open and filled boxes represent the putative σ^54^-dependent promoter and upstream activator sequences, respectively.

In our previous study, a group of genes (PST1302 to PST1359) located contiguously in the A1501 genome was defined as a nitrogen fixation island [20]. This cluster represents the largest assembly of genes responsible for the synthesis, maturation, and function of the nitrogenase complex that has been characterized in any free-living diazotrophic species to date. Genes in this region had the highest transcription levels (>10-fold compared to almost all the *nif *genes) under nitrogen fixation conditions and were remarkably downregulated by ammonium shock (Figure 2A, Table 1). These results, together with the observation that genes outside the island are downregulated under nitrogen fixation conditions (Figure 2A), indicate that the nitrogen fixation island functions to support nitrogen fixation. Similar findings were reported in a previous study in which expression islands were shown to be clustered on a large symbiosis island of the *M. loti *genome [8].

**Table 1:** Transcriptional characteristics of the genes within the nitrogen fixation island of *P. stutzeri *A1501

	Gene name	Functional description	Ammonium shock^a^	Nitrogen fixation^b^	Consensus sequence^c^	
					**NifA**	**RpoN**

PST1302		glutaredoxin-related protein	0.06	16.83		
PST1303		thiosulfate sulfurtransferase glpE	0.03	53.99		
PST1304	*nifQ*	nitrogen fixation protein NifQ	0.03	46.56		
PST1305		arsenate reductase related protein	0.03	38.67		
PST1306	*nifB*	FeMo cofactor biosynthesis protein NifB	0.06	21.46	Yes	Yes

PST1307		conserved hypothetical protein	0.57	1.87	No	Yes

PST1308		transcriptional regulator, LysR family	0.43	2.22	No	No

PST1309		conserved hypothetical protein	0.84	1.12	No	No

PST1310		transcriptional regulator, AraC family	1.03	1.02	No	No

PST1311		fosmidomycin resistance protein	0.81	1.34	No	Yes

PST1312	*tpmA*	thiopurine s-methyltransferase	0.34	2.65	No	Yes

PST1313	*nifA*	positive regulatory protein	0.15	6.95		
PST1314	*nifL*	negative regulatory protein	0.12	7.68	Yes	Yes

PST1315	*rnfA*	electron transport complex, A subunit	0.37	2.66	Yes	Yes
PST1316	*rnfB*	electron transport complex, B subunit	0.10	9.94		
PST1317	*rnfC*	electron transport complex, C subunit	0.49	2.22		
PST1318	*rnfD*	electron transport complex, D subunit	0.13	7.67		
PST1319	*rnfG*	electron transport complex, G subunit	0.16	7.47		
PST1320	*rnfE*	electron transport complex, E subunit	0.15	8.80		
PST1321	*rnfH*	electron transport complex, H subunit	0.21	17.55		
PST1322	*nifY2*	iron-molybdenum cofactor biosynthesis protein	0.10	21.74		
PST1323		nitrogen fixation-related protein	0.18	13.73		

PST1324		conserved hypothetical protein	0.15	25.99		
PST1325^#^		conserved hypothetical protein	0.15	9.68	Yes	Yes

PST1326	*nifH*	Fe protein	0.32	94.05	Yes	Yes
PST1327	*nifD*	MoFe protein, alpha subunit	0.56	54.16		
PST1328	*nifK*	MoFe protein, beta subunit	0.62	38.22		
PST1329	*nifT*	nitrogen fixation protein	1.70	7.82		
PST1330	*nifY*	iron-molybdenum cofactor biosynthesis protein	0.33	8.51		
PST1331		conserved hypothetical protein	0.41	12.55		

PST1332		leucine-rich repeat domain protein	0.57	3.27	No	Yes

PST1333	*nifE*	iron-molybdenum cofactor biosynthesis protein	0.05	35.82	Yes	Yes
PST1334	*nifN*	iron-molybdenum cofactor biosynthesis protein	0.07	13.32		
PST1335	*nifX*	iron-molybdenum cofactor biosynthesis protein	0.10	37.97		
PST1336		protein of unknown function DUF269	0.29	5.06		
PST1337		protein of unknown function DUF683	0.05	63.84		

PST1338		ferredoxin, 4Fe-4S	0.08	31.07	Yes	Yes
PST1339		ferredoxin, 2Fe-2S	0.76	2.94		
PST1340		conserved hypothetical protein	0.82	1.28		
PST1341		conserved hypothetical protein	0.85	1.28		
PST1342		conserved hypothetical protein	0.43	3.69		
PST1343		conserved hypothetical protein	0.70	2.56		

PST1344		conserved hypothetical protein	0.21	7.16	Yes	Yes

PST1345	*modC*	molybdenum transport protein ModC	0.79	1.59		
PST1346	*modB*	molybdate ABC transporter	0.48	2.05		
PST1347	*modA*	molybdenum ABC transporter	0.20	4.12		
PST1348		putative molybdenum-binding protein	0.22	3.88	Yes	Yes

PST1349	*hesB*	Fe-S cluster assembly protein	0.07	20.92	Yes	Yes
PST1350	*nifU*	Fe-S cluster assembly protein NifU	0.11	10.77		
PST1351	*nifS*	nitrogenase metalloclusters biosynthesis protein	0.11	16.21		
PST1352	*nifV*	homocitrate synthase	0.05	24.55		
PST1353	*cysE*	serine acetyltransferase (cysE-like)	0.05	32.98		
PST1354		conserved hypothetical protein	0.10	11.11		
PST1355	*nifW*	nitrogenase stabilizing/protective protein	0.16	26.04		
PST1356	*nifZ*	Fe-S cofactor synthesis protein	0.17	18.17		
PST1357	*nifM*	putative peptidyl-prolyl cis/trans isomerase	0.19	18.78		
PST1358		ATP-dependent Clp protease	0.56	8.42		

PST1359	*nifF*	flavodoxin for electron transfer	0.86	14.91	Yes	Yes

^a^Induction ratio (ammonium shock conditions/nitrogen fixation conditions) after exposure of bacteria to 20 mM ammonia for 10 min.

^b^Induction ratio (nitrogen fixation conditions/nitrogen-excess conditions).

^c^ORF(s) preceded by a consensus sequence that resembles the NifA or RpoN consensus sequence for A1501. The predicted NifA-σ^54^-dependent operons are shown in Figure 2B.

^#^Also designated *pnfA *in this study.

The σ^54 ^factor (or RpoN) is a well-known alternative factor for RNA polymerase that enables the transcription of *nif *genes in conjunction with the transcriptional activator NifA or activates other genes involved in nitrogen metabolism and other functions with NtrC, PspF, DctD [23]. To identify putative σ^54^-dependent operons, the promoter regions of the regulon genes induced specifically under nitrogen fixation conditions were searched *in silico *for the presence of a -24/-12 promoter sequence. As anticipated, a very high proportion of the regulon genes had a σ^54 ^consensus promoter (Additional file 3). This result is in agreement with the fact that σ^54 ^is a global regulator that is involved in a complex transcriptional network that controls a large variety of cellular processes [24,25]. As described above, the expression of the *nif *genes is known to be controlled by a common mechanism in diazotrophic Proteobacteria. In this mechanism, NifA recognizes an upstream activator sequence (TGT-N_10_-ACA) usually located at least 100-bp upstream of the transcription initiation site [26,27]. Promoter sequence analysis of genes within the island indicated that a total of 52 genes are organized into 11 putative NifA-σ^54^-dependent operons (Figure 2B). These include the *nifLA *cluster, which encodes negative and positive regulators; the *rnfABCDGEF *cluster, which encodes the subunits of a membrane-bound protein complex involved in electron transport to nitrogenase; the *nifHDK *cluster, which encodes dinitrogenase reductase (NifH) and dinitrogenase (NifDK); the *nifENX *cluster, which is involved in the synthesis of the iron-molybdenum cofactor of nitrogenase (FeMo-co); and the molybdate transport genes *modAB*. In order to substantiate the *in silico *predictions of the NifA-σ^54^-dependent promoters, we compared transcriptional level expression of the 11 operons between the wild type strain and the *nifA *or *rpoN *mutants by quantitative RT-PCR. Under nitrogen fixation conditions, the expression of the 11 predicted NifA-σ^54^-dependent operons was upregulated up to 10-fold in the wild type strain but was almost completely repressed in the *nifA *and *rpoN *mutants (data not shown). Our data indicate that the 11 operons in the island are activated in a NifA-σ^54^-dependent manner under nitrogen fixation conditions.

### Inactivation of selected nitrogen fixation-inducible genes

Biological nitrogen fixation is an oxygen-sensitive and energy-dependent process that requires ATP and a supply of Fe-S clusters not only for the nitrogenase components but also for many other proteins involved in electron transfer, redox and non-redox catalysis, and sensing of regulatory processes. Our previous study demonstrated that mutation of 16 genes within the nitrogen fixation island led to a decrease in nitrogenase activity. However, the role of these genes in nitrogen fixation remained hypothetical due to the lack of further genetic and physiological characterization [20]. In the present study, we performed a global transcriptional profiling analysis of nitrogen fixation and ammonium repression in A1501 and identified a regulon of genes induced specifically under nitrogen fixation conditions. Among the regulon genes, 52 were located in the nitrogen fixation island, whereas 114 were located outside the island. Many of the regulon genes encode previously uncharacterized proteins, such as transcriptional regulators or outer membrane proteins. To identify their physiological roles, we inactivated nine regulon genes by homologous suicide plasmid integration. The nitrogenase activities of the corresponding mutant strains were determined and are summarized in Table 2. Mutation of PST3417 and PST4084, which encode a predicted transcriptional regulator and a predicted signal transduction protein, respectively, led to a significant decrease in nitrogenase activity. PST0874 encodes a predicted chemotactic transducer. We constructed a mutant that carried a defective PST0874 gene and found that the mutant strain displayed 57% of intact wild type nitrogenase activity (Table 2). Our results suggest that these previously uncharacterized genes, although not essential, are required for full nitrogenase activity. However, the possible role of these genes in electron transfer, redox and non-redox catalysis, and sensing of regulatory processes requires further investigation.

**Table 2:** Identification of nine selected nitrogen fixation-inducible genes and nitrogenase activity of the corresponding mutant strains

Gene ID	Functional description	Involvement in nitrogen fixation in other systems	Nitrogenase activity^a^, %
PST0446	Cytoplasmic membrane protein	Unknown	64
PST0874	Chemotactic transducer	Unknown	52
PST1325	conserved hypothetical protein	Unknown	23
PST1521	outer membrane protein	Unknown	68
PST2508	methyl-accepting chemotaxis transducer	Unknown	92
PST3417	predicted transcriptional regulators	Unknown	58
PST3422	outer membrane protein	Unknown	96
PST3621	transcriptional regulator, AraC family	Unknown	80
PST4084	ABC-type amino acid transport/signal transduction systems	Unknown	54

^a^Nitrogenase activity of nine non-polar mutants (enabling transcription of downstream genes) is expressed as % of wild type A1501 activity, which is in the range of 1645 ± 169 nmol ethylene/min/mg protein. Nitrogenase activity was examined under nitrogen fixation conditions (0.1 mM ammonium and 0.5% oxygen tension). Data are expressed as the means of three independent experiments.

### Functional analysis of a new nitrogen fixation gene

**Figure 3:**
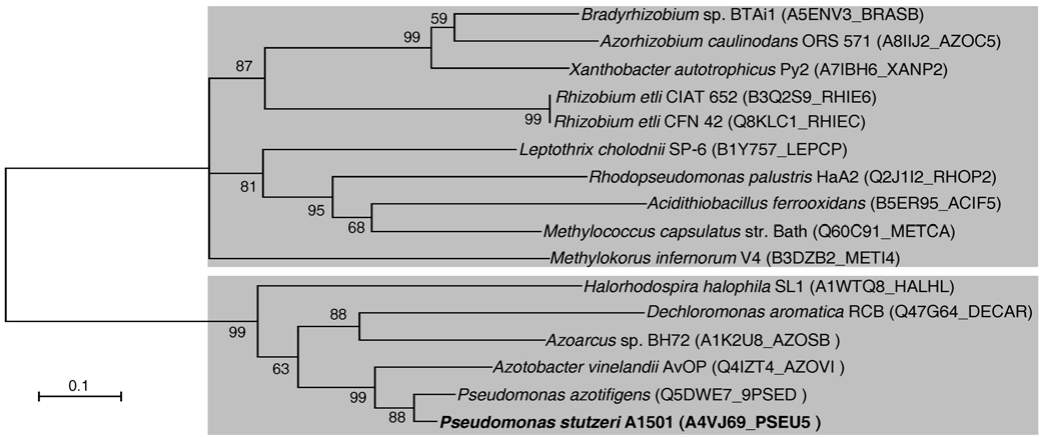
**Unrooted neighbor-joining phylogenetic tree of the A1501 PnfA and related proteins from other bacteria**. Bootstrap values based on 1,000 replications are listed as percentages at branching points. Bar, 0.1 substitutions per position. The sequence abbreviations are given in parentheses.

**Figure 4:**
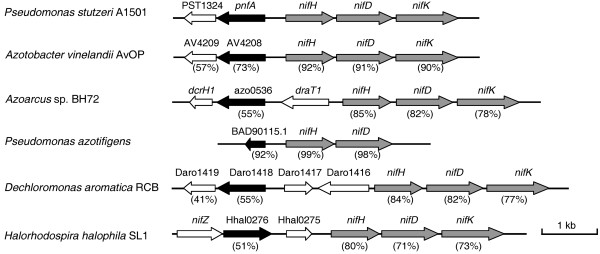
**Physical organization of the A1501 *pnfA *cluster and comparisons with equivalent clusters from other diazotrophic strains**. The numbers underneath the arrows indicate the percentage of amino acid sequence identity between the A1501 *pnfA *product and its homologs.

**Figure 5:**
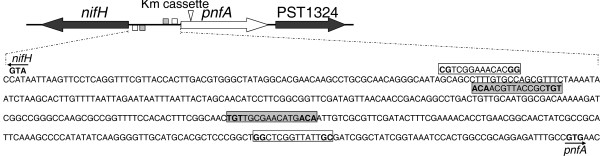
**Intergenic region between *nifA *and *pnfA *and construction of the *pnfA *non-polar mutant A1325**. The open and filled boxes represent the putative σ^54^-dependent promoter and upstream activator sequences, respectively. The inverted triangle indicates the location of the mutations inserted into *pnfA *via homologous suicide plasmid integration.

**Figure 6:**
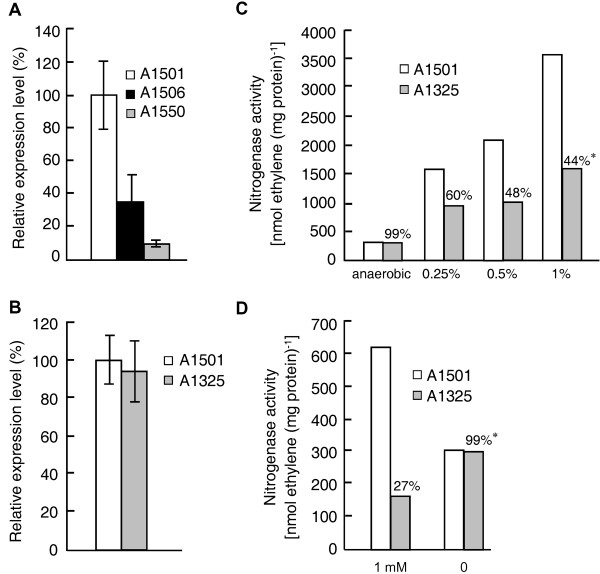
**Phenotypic analyses of the mutation inserted into the *pnfA *gene**. (A) Quantitative RT-PCR analysis of *pnfA *expression in the wild type A1501, *nifA *mutant A1506, and *rpoN *mutant A1550 strains under nitrogen fixation conditions. (B) Quantitative RT-PCR analysis of *nifH *expression in the wild type A1501 and mutant A1325 strains under nitrogen fixation conditions. (C) The effect of different oxygen concentrations on nitrogenase activity in the wild type A1501 and mutant A1325 strains. (D) Nitrogenase activity in the wild type A1501 and mutant A1325 strains grown under anaerobic conditions with or without 1 mM nitrate. * The nitrogenase activity of the mutant strain is expressed as a percentage (%) of the wild type A1501 activity. Results shown are representative of four (C) or three (D) independent experiments.

On the basis of the above observations, we speculate that some as yet unknown genes may be involved in nitrogen fixation. Upon searching for such genes, a new NifA-σ^54^-dependent gene, termed *pnfA*, was identified. Phylogenetic analysis suggested that the A1501 PnfA protein and related proteins in other microorganisms can be classified into two distinct subfamilies (Figure 3). The PnfA protein and its five homologs comprised the first subfamily. Ten hypothetical proteins, with 20% identity to the A1501 PnfA, comprised the second subfamily. The A1501 *pnfA *gene encodes a 31.7-kDa protein with high amino acid sequence similarity to five hypothetical proteins found only in diazotrophic strains, namely *P. azotifigens *(92% identity), *A. vinelandii *AvoP (72% identity), *Azoarcus *sp. BH72 (55% identity), *Dechloromonas aromatica *RCB (54% identity), and *Halorhodospira halophila *SL1 (49% identity), suggesting that it may be a diazotroph-specific protein. The *pnfA *gene is chromosomally linked to *nifHDK *and is transcribed in the opposite orientation (Figure 4). Sequence analysis of the *nifH-pnfA *intergenic region revealed a putative -24/-12 promoter sequence and the upstream activator sequence TGT-N_10_-ACA (Figure 5). These data, together with the experimental evidence obtained from quantitative RT-PCR analysis, led us to conclude that *pnfA *is activated in a NifA-σ^54^-dependent manner, a characteristic feature for activation of the well-known *nif *genes under nitrogen fixation conditions (Figure 6A).

To investigate its biological function, a non-polar mutant of *pnfA *was constructed, and the effect of this mutation on the expression and activity of nitrogenase under nitrogen fixation conditions was studied. No significant difference in the transcriptional levels of *nifH *was observed between the wild type and *pnfA *mutant under nitrogen fixation conditions, suggesting that PnfA does not have any regulatory role in the transcription of *nifHDK *(Figure 6B). Complementation of the *pnfA *mutant with pVK100-1325, in which the expression of *pnfA *was monitored from the *km *promoter, restored the activity of nitrogenase to wild type levels (data not shown). However, the non-polar mutant strain displayed much lower nitrogenase activity than the wild type strain under nitrogen fixation conditions (0.1 mM ammonium and 0.5% oxygen tension) (Figure 6C), suggesting that PnfA is necessary for maximal nitrogenase activity.

To further determine the function of the new gene, we determined the effects of PnfA inactivation on nitrogenase activity under different oxygen concentrations, especially under anaerobic conditions using nitrate as the terminal electron acceptor. The incubation chamber was thoroughly flushed with pure argon gas to generate an anaerobic environment. As shown in Figure 6C, the activity of wild type nitrogenase was much lower under anaerobic conditions than under microaerobic conditions. In contrast to its activity under microaerobic conditions, the nitrogenase activity of mutant A1325 was similar to that observed in the wild type strain under anaerobic conditions, suggesting that PnfA regulates the activity of nitrogenase under miocroaerobic but not anaerobic conditions. Upon increasing the concentration of oxygen from 0 to 1%, a remarkable decrease in the percentage of nitrogenase activity was observed in the mutant and wild type strains (Figure 6C). Note that the nitrogenase activity of A1501 led to the production of approximately 600 μmol ethylene/mg of protein when the bacteria were grown under anaerobic conditions in nitrogen-free medium supplemented with 1 mM nitrate (Figure 6D). This activity was much higher than that observed for the *pnfA *mutant. Obviously, the mutant loses its ability to fix nitrogen under anaerobic conditions when nitrate serves as the terminal electron acceptor. Overall, these results, together with the finding that PnfA contains an amino acid sequence with approximately 31% identity to the equivalent region from the universal stress protein A or the electron transfer flavoprotein alpha subunit-like protein, led us to conclude that the PnfA protein is involved in nitrogen fixation processes, such as electron transfer to nitrogenase or oxygen protection mechanisms.

## Discussion

Nitrogen fixation is tightly regulated at the transcriptional and post-translational levels [2]. The first checkpoint occurs at the level of the *nif*-specific NifA-NifL interaction, which is controlled by the Ntr systems [28]. The second checkpoint occurs upon reversible inactivation of nitrogenase by dinitrogenase reductase ADP-ribosyltransferase (DraT) and dinitrogenase reductase glycohydrolase (DraG) [29]. In many diazotrophic γ-Proteobacteria, such as in *K. pneumoniae*, *A. vinelandii*, and *P. stutzeri*, the σ^54^-dependent activator NifA activates the expression of all the *nif *genes, and the anti-activator NifL controls NifA activity via protein-protein interactions in response to fixed nitrogen status [18,30,31]. Different regulatory mechanisms at the transcriptional level have been documented in several diazotrophs, such as in the α-Proteobacteria *A. brasilense*, which lacks *nifL *[32]. PII proteins are key signal transduction proteins involved in the general nitrogen regulation system and are found in diazotrophic Proteobacteria as two paralogous gene copies, *glnB *and *glnK *[19,33-35]. In *K. pneumoniae*, the GlnB protein controls the activity of the NtrB-NtrC regulatory system; in turn, phosphorylation of NtrC controls the expression of the alternative PII protein GlnK, as well as of the NifL and NifA proteins [28]. A1501 carries a single copy of the *glnK *gene, similar to *A. vinelandii*, but lacks *glnB*, which is present in *K. pneumoniae *and *A. brasilense *[28,32]. GlnK can interact with NifL to promote the formation of a GlnK-NifL-NifA ternary complex in *A. vinelandii *and *P. stutzeri *[19,36]. Here we report a global transcriptional profiling analysis of nitrogen fixation and ammonium repression in A1501 using a whole-genome DNA microarray. The results show that among the nitrogen fixation-inducible genes in A1501, including those involved in the Ntr and *nif*-specific regulatory systems, 95 have orthologs in *Azoarcus *sp. BH72 and *A. vinelandii *AvoP. This type of detailed global analysis had not been done before. Our results suggest the existence of similar regulatory mechanisms that control expression of nitrogen fixation genes in the three most closely related diazotrophic strains. Moreover, although nitrogenase on-off switching was previously reported [13], DraT and DraG (known to control nitrogenase ADP ribosylation) have not been found in A1501, suggesting that it possesses an inactivation mechanism different from that reported in *Rhodospirillum rubrum *[37], *Azoarcus *[38], and *A. brasilense *[39].

Horizontal gene transfer plays an important role in the evolution of the nitrogen fixation system [40-43]. Baar *et al*. [44] observed that a gene cluster containing the *nif *genes within the genome of *Wolinella succinogenes *exhibits a high degree of synteny to the cyanobacterial *nif *gene cluster, implying that the *nif *genes may have been acquired by gene transfer. A so-called "symbiosis island" was initially identified in *M. loti *ICMP3153 as a transmissible 500-kb DNA element, which contains all the genes that are likely to be required for Nod factor, nitrogen fixation, and island transfer [45]. After years of controversy, it is now established that several strains of *Pseudomonas *species can fix nitrogen [14]. However, the sporadic occurrence of functional *nif *genes in *Pseudomonas *spp. raises the question of their origin [16]. Based on genomic comparisons, the number of genes previously recognized as being part of the *Pseudomonas *core genome may be limited to 1,997 [20]. In the present study, we found that more than 70% of nitrogen fixation-inducible genes, including those within the nitrogen fixation island, are absent from the *Pseudomonas *core genome. Only 24 nitrogen fixation-inducible genes, such as the *ntr *genes, belong to the *Pseudomonas *core genome (Table 3). Our data on global transcriptional profiling strongly suggest that the A1501 *nif *region is not only a genomic island but also an expression island. The *ntr *system exists in almost all organisms, whereas the *nifLA *regulatory system is highly specific for nitrogen-fixing bacteria [33,34]. Apparently, A1501 has its own *ntr *systems and acquired a *nif*-specific regulatory system from a diazotrophic ancestor during its evolution. Consequently, the acquisition of *nif *genes and cooperative control of two regulatory systems of different evolutionary origins confer A1501 with the unusual capability to fix nitrogen. However, the consequences of *nif *island acquisition for the host and for island function upon entrance into a new global regulatory network have not been directly studied.

The rhizosphere is a densely populated area and exerts a unique influence on microbial activity. Associative nitrogen fixation is often enhanced in the rhizosphere compared with bulk soil, because root-associated diazotrophs can utilize more root exudates as carbon and energy sources. The A1501 genome contains genes likely to be involved in broad utilization of carbon sources, nitrogen fixation, denitrification, degradation of aromatic compounds, the regulation of multiple pathways involved in protection against environmental stresses, and other functions that presumably give A1501 an advantage in the rhizosphere [20]. The ability to fix nitrogen is compatible with a wide range of physiologies including aerobic (for example *A. vinelandii*), microaerobic (for example *P. stutzeri*) or anaerobic (for example *K. pneumoniae*) heterotrophs [2]. The physiology of nitrogen fixation is more restricted in *K. pneumoniae *than in *P. stutzeri *and *A. vinelandii*.

**Table 3:** Nitrogen fixation-inducible genes that belong to the *Pseudomonas *core genome

Gene ID	Gene name	Functional description
PST0200		4-hydroxyphenylpyruvate dioxygenase
PST0349	*ntrC*	nitrogen regulation protein NtrC
PST0350	*ntrB*	nitrogen regulation protein NtrB
PST0353	*glnA*	glutamine synthetase
PST0502	*glnK*	nitrogen regulatory protein P-II
PST0503	*amtB1*	ammonium transporter
PST0722	*prkA*	serine protein kinase PrkA
PST0754		membrane protein
PST0813		major facilitator family transporter
PST1140		conserved hypothetical protein
PST1301	*cobs*	cobalamin (5'-phosphate) synthase
PST1346	*modB*	molybdate ABC transporter, permease protein
PST1347	*modA*	molybdenum ABC transporter, periplasmic binding protein
PST1563	*adhC*	alcohol dehydrogenase class III
PST2137	*glgA*	glycogen synthase
PST2154		alpha-amylase family protein
PST2381		conserved hypothetical protein
PST2748		OmpA family protein
PST2982	*braC*	branched-chain amino acid transport protein BraC
PST3253		membrane protein, putative
PST3727	*urea*	urease, gamma subunit
PST3736	*ureE*	urease accessory protein UreE
PST3780	*rodA*	rod-shape-determining protein RodA
PST3795		ribosome-associated GTPase

We further compared the organization of the A1501 *nif *cluster with that of *K. pneumoniae*, which exhibits the highest organization of *nif *genes identified to date. The high conservation of the core *nif *genes between the two diazotrophic strains suggests that they were acquired from a common ancestor. Interestingly, we found that many additional genes, such as *pnfA*, which is associated with the core *nif *genes, are found in A1501 but not exist in *K. pneumoniae*. From these facts, we suppose that lateral transfer of the *nif *genes from a common ancestor and subsequent acquisition of additional genes occurred to for bacterial adaptation to the physiology of their respective hosts. In nitrogen-free semisolid medium, A1501 formed a pellicle under the surface and exhibited a high level of nitrogenase activity, suggesting that nitrogen fixation occurs at low oxygen tension [13]. Under anaerobic conditions, nitrate can accept the terminal electron transferred from the respiratory chain, and the energy generated during this process supports nitrogen fixation in A1501 [46]. However, the genes responsible for the transport of electrons to nitrogenase and for the oxygen protection mechanisms in A1501 have yet to be determined.

The objective of this study was to explore the utility of transcriptional profiling as a screening method for identifying previously uncharacterized genes relevant for the nitrogen fixation processes. In addition, we focused on elucidating the roles of newly characterized genes in controlling the expression or activity of nitrogenase. We first identified a set of hypothetical genes that are induced under nitrogen fixation conditions and subsequently carried out a functional analysis of these genes. Consequently, we identified a new gene, referred to as *pnfA*, which is chromosomally linked to *nifHDK*. This new gene has the following characteristics: (i) like all known *nif *genes, it is upregulated exclusively under nitrogen fixation conditions in a NifA-σ^54^-dependent manner; (ii) it encodes a 31.7-kDa protein with high similarity to hypothetical proteins present only in nitrogen-fixing bacteria, suggesting that it is a diazotroph-specific protein; (iii) its inactivation results in the inability of bacteria to use nitrate as a terminal electron acceptor for supporting nitrogen fixation under anaerobic conditions. However, the *pnfA *mutant can grow on nitrogen-free semisolid medium, indicating that it has a Nif^+ ^phenotype (data not shown). Furthermore, a similarity search revealed that a portion of the amino acid sequence of the PnfA protein (residues 176 to 240) has approximately 31% identity to equivalent regions from the UspA domain-containing protein of *Mycobacterium *sp. JLS and the electron transfer flavoprotein alpha subunit-like protein of *Methylobacterium nodulans *ORS 2060. This result suggests that PnfA may play a role in the transport of electrons to nitrogenase or in the regulation of oxygen protection mechanisms. This finding is also supported by the observation that homologues of PnfA are present in many associative or endophytic nitrogen-fixing bacteria, such as A1501, *Azoarcus sp*. BH72, and *A. vinelandii *AvoP, which usually utilize different strategies for meeting the energy demand of nitrogen fixation and protecting nitrogenase from oxygen. Further studies on the expression and regulation of nitrogen fixation-inducible genes in A1501 will provide useful insights into how the bacterium responds to environmental cues and how it adapts to rhizosphere environments.

## Conclusions

In the present study, we performed a global transcriptional profiling analysis of nitrogen fixation and ammonium repression in *P. stutzeri *A1501 using a whole-genome DNA microarray. In total, 166 genes were upregulated under nitrogen fixation conditions and rapidly downregulated as early as 10 min after ammonium shock, suggesting the existence of a regulon of genes that are induced specifically under nitrogen fixation conditions. Strikingly, we found that a 49-kb cluster of all *nif *and associated genes was markedly upregulated as an expression island, whereas genes outside the island were downregulated under nitrogen fixation conditions. Furthermore, we identified a new NifA-σ^54^-dependent gene, termed *pnfA*, which is located within the expression island and may be involved in the nitrogen fixation processes. The implications of our functional and evolutionary analyses of the nitrogen fixation island and the *pnfA *gene in root-associated and nitrogen-fixing bacteria are discussed.

## Methods

### Bacterial strains and growth conditions

*P. stutzeri *A1501 and the mutant derivatives were grown at 30°C in Luria-Bertani medium or in minimal lactate-containing medium (medium K) as described previously [13]. Kanamycin (Km) was added to the medium at a concentration of 50 μg/mL as required. A1501 was cultured under two growth conditions: nitrogen fixation conditions (0.1 mM ammonium and 0.5% oxygen tension) and nitrogen-excess conditions (20 mM ammonium and 0.5% oxygen tension). For expression assays, A1501 was exposed to ammonium shock for 10 min, which involved a sudden change from nitrogen fixation to ammonium shock conditions by addition of 20 mM ammonium. We verified that no discernible loss of ammonium occurred after the shock. The activity of nitrogenase in A1501 is completely repressed by 20 mM ammonium.

### Microarray analysis

Microarray fabrication, cDNA synthesis, labeling, hybridization, and analysis of the data were conducted as described previously [20].

### Quantitative real-time PCR analysis

Quantitative RT-PCR experiments were performed as described previously [20] and according to the manufacturer's recommendations using the RG6000 Q-PCR thermocycler (Corbett Research, Mortlake, NSW, Australia) and the SYBR Green PCR Master Mix (Applied Biosystems, Foster City, CA).

### Construction of non-polar mutants

Uncharacterized genes were inactivated by homologous suicide plasmid integration as described previously [47] using pK18 mob as the vector [48].

### Nitrogenase activity assays

To examine the activity of nitrogenase, bacterial suspensions were incubated in N-free minimal medium at an OD_600 _of 0.1 at 30°C under an argon atmosphere containing oxygen at different concentrations and acetylene at 10% according to the protocol described by Desnoues *et al*. [13]. After the concentration of oxygen was adjusted to 0, 0.25, 0.5, and 1.0%, acetylene was injected, and gas samples were withdrawn periodically for gas chromatographic analysis of ethylene production. Under anaerobic conditions, the activity of nitrogenase was analyzed by incubating bacterial suspensions in N-free minimal lactate medium supplemented with 1 mM nitrate at an OD_600 _of 0.1. The specific activity of nitrogenase was expressed as nmol ethylene/min/mg protein. Protein concentrations were determined using a standard protein assay (Bio-Rad, Hercules, CA) with bovine serum albumin as the standard. Each experiment was repeated at least three times.

### Microarray data accession number

The gene expression data have been deposited in the Gene Expression Omnibus (GEO) database under the accession numbers GSE14775 and GSE6572.

## Authors' contributions

YY and SP performed the microarray experiments, analyzed the gene expression data, and helped write the manuscript. YD and MC performed microarray experiments and analyzed the data. YH, LL, DL and YZ constructed the mutants. YL, HF and YF measured the activity of nitrogenase. JP and JY helped with the initial microarray analyses. WZ and WL performed the qPCR experiments. QC and QJ designed the experiments. ML designed experiments, analyzed data, and helped write the manuscript. All authors read and approved the final manuscript.

## Supplementary Material

Additional file 1 **Upregulation (a) or downregulation (b) of genes in *P. stutzeri *A1501 grown under nitrogen fixation vs. nitrogen-excess conditions**. Click here for file

Additional file 2 **Upregulation (a) or downregulation (b) of genes 10 minutes after exposure of bacteria to ammonium shock**. Click here for file

Additional file 3 **Genes induced specifically under nitrogen fixation conditions**. Click here for file
