# Supplementary material for: Global transcriptional analysis of nitrogen fixation and ammonium repression in root-associated Pseudomonas stutzeri A1501
Source: BMC Genomics. 2010 Jan 7;11:11. doi: 10.1186/1471-2164-11-11 (PMC2820453; doi:10.1186/1471-2164-11-11)
Supplement: Additional file 1 — Upregulation (a) or downregulation (b) of genes in P. stutzeri A1501 grown under nitrogen fixation vs. nitrogen-excess conditions. [file 1471-2164-11-11-S1.PDF]

Additional file 1

Upregulation (a) or downregulation (b) of genes in *P. stutzeri* A1501 grown under nitrogen fixation vs. nitrogen-excess conditions

Number	Gene ID	Gene name	Microarray ratio	Functional description
(a) Upregulated genes				
1	PST0029		3.45	conserved hypothetical protein
2	PST0035		3.74	coserved hypothetical protein
3	PST0039	*katE*	2.27	catalase
4	PST0167		2.37	formate dehydrogenase-O, major subunit
5	PST0179	*ectC*	2.06	L-ectoine synthase
6	PST0200		3.82	4-hydroxyphenylpyruvate dioxygenase
7	PST0241		2.3	arsenic resistance transcriptional regulator
8	PST0265	*osmC*	3.86	osmotically inducible protein OsmC
9	PST0266		4.16	ribonucleotide reductase, alpha subunit
10	PST0349	*ntrC*	3.19	nitrogen regulation protein NtrC
11	PST0350	*ntrB*	2.86	nitrogen regulation protein NtrB
12	PST0353	*glnA*	2.26	glutamine synthetase
13	PST0368		2.42	conserved hypothetical protein
14	PST0387		2.09	conserved hypothetical protein
15	PST0446		2.57	cytoplasmic membrane protein
16	PST0502	*glnK*	6.85	nitrogen regulatory protein P-II
17	PST0503	*amtB1*	3.86	ammonium transporter
18	PST0504	*amtB2*	2.55	ammonium transporter
19	PST0565		2.4	major facilitator family transporter
20	PST0571		2.24	conserved hypothetical protein
21	PST0577		2.11	conserved hypothetical protein
22	PST0579		2.4	GMP synthase, PP-ATPase domain/subunit
23	PST0585		2.3	site-specific recombinase, phage integrase family
24	PST0586		2.84	conserved hypothetical protein
25	PST0610		2.05	dihydroxyacid dehydratase/phosphogluconate dehydratase
26	PST0632		2.51	type I restriction-modification system, S subunit
27	PST0692	*phaP*	2.05	phasin PhaP
28	PST0721		2.37	conserved hypothetical protein
29	PST0722	*prkA*	2.07	serine protein kinase PrkA
30	PST0752		2.03	membrane protein
31	PST0754		4.19	membrane protein
32	PST0757		2.04	peptidase, M23/M37 family

33	PST0811	*katA*	2.17	catalase
34	PST0813		3.29	major facilitator family transporter
35	PST0856		3.08	conserved hypothetical protein
36	PST0874	*pctA*	2.11	chemotactic transducer PctA
37	PST0937		2.44	conserved hypothetical protein
38	PST0949		2.21	conserved hypothetical protein
39	PST0970	*pilY1*	2.04	type 4 fimbrial biogenesis protein PilY1
40	PST0972	*pilW*	2.5	type 4 fimbrial biogenesis protein PilW
41	PST0984		2.12	lipoprotein, putative
42	PST1000		2.4	putative membrane protein
43	PST1054	*pilA*	2.16	fimbrial protein ecpC precursor
44	PST1140		2.84	conserved hypothetical protein
45	PST1169	*algA*	2.42	phosphomannose isomerase
46	PST1273		2.13	putative membrane protein
47	PST1279		2.42	conserved hypothetical protein
48	PST1301	*cobS*	8.97	cobalamin (5'-phosphate) synthase
49	PST1302		16.83	glutaredoxin-related protein
50	PST1303		53.99	thiosulfate sulfurtransferase glpE
51	PST1304	*nifQ*	46.56	nitrogen fixation protein NifQ
52	PST1305		38.67	arsenate reductase related protein
53	PST1306	*nifB*	21.46	FeMo cofactor biosynthesis protein NifB
54	PST1308		2.22	transcriptional regulator, LysR family
55	PST1312	*tpmA*	2.65	thiopurine s-methyltransferase
56	PST1313	*nifA*	6.95	nitrogen fixation positive regulatory protein
57	PST1314	*nifL*	7.68	nitrogen fixation negative regulatory protein
58	PST1315	*rnfA*	2.66	electron transport complex, RnfABCDGE type, A subunit
59	PST1316	*rnfB*	9.94	electron transport complex, RnfABCDGE type, B subunit
60	PST1317	*rnfC*	2.22	electron transport complex, RnfABCDGE type, C subunit
61	PST1318	*rnfD*	7.67	electron transport complex, RnfABCDGE type, D subunit
62	PST1319	*rnfG*	7.47	electron transport complex, RnfABCDGE type, G subunit
63	PST1320	*rnfE*	8.8	electron transport complex, RnfABCDGE type, E subunit
64	PST1321	*rnfH*	17.55	electron transport complex, RnfABCDGE type, H subunit
65	PST1322	*nifY2*	21.74	dinitrogenase iron-molybdenum cofactor biosynthesis
66	PST1323		13.73	nitrogen fixation-related protein
67	PST1324		25.99	conserved hypothetical protein
68	PST1325		9.68	conserved hypothetical protein
69	PST1326	*nifH*	94.05	Fe protein, nitrogenase reductase NifH
70	PST1327	*nifD*	54.16	MoFe protein, alpha subunit
71	PST1328	*nifK*	38.22	MoFe protein, beta subunit
72	PST1329	*nifT*	7.82	nitrogen fixation protein
73	PST1330	*nifY*	8.51	nitrogenase iron-molybdenum cofactor biosynthesis

74	PST1331		12.55	conserved hypothetical protein
75	PST1332		3.27	leucine-rich repeat domain protein
76	PST1333	*nifE*	35.82	nitrogenase iron-molybdenum cofactor biosynthesis protein
77	PST1334	*nifN*	13.32	nitrogenase iron-molybdenum cofactor biosynthesis protein
78	PST1335	*nifX*	37.97	nitrogenase iron-molybdenum cofactor biosynthesis protein
79	PST1336		5.06	protein of unknown function DUF269
80	PST1337		63.84	protein of unknown function DUF683
81	PST1338		31.07	ferredoxin, 4Fe-4S
82	PST1339		2.94	ferredoxin, 2Fe-2S
83	PST1342		3.69	conserved hypothetical protein
84	PST1343		2.56	conserved hypothetical protein
85	PST1344		7.16	conserved hypothetical protein
86	PST1346	*modB*	2.05	molybdate ABC transporter, permease protein
87	PST1347	*modA*	4.12	molybdenum ABC transporter, periplasmic binding protein
88	PST1348		3.88	putative molybdenum-binding protein
89	PST1349	*hesB*	20.92	Fe-S cluster assembly protein
90	PST1350	*nifU*	10.77	Fe-S cluster assembly protein NifU
91	PST1351	*nifS*	16.21	nitrogenase metalloclusters biosynthesis proteinNifS
92	PST1352	*nifV*	24.55	NifV protein, encodes a homocitrate synthase
93	PST1353	*cysE*	32.98	serine acetyltransferase (cysE-like)
94	PST1354		11.11	conserved hypothetical protein
95	PST1355	*nifW*	26.04	nitrogenase stabilizing/protective protein nifW
96	PST1356	*nifZ*	18.17	Fe-S cofactor synthesis protein
97	PST1357	*nifM*	18.78	NifM protein, putative a peptidyl-prolyl cis/trans isomerase
98	PST1358		8.42	ATP-dependent Clp protease, ATP-binding subunit ClpX
99	PST1359	*nifF*	14.91	flavodoxin required for electron transfer to the Fe protein
100	PST1360		7.27	glutathione peroxidase
101	PST1379	*algZ*	2.85	alginate biosynthesis transcriptional activator
102	PST1380		2.55	conserved hypothetical protein
103	PST1404	*fliC*	3.1	flagellin type B
104	PST1471	*hsdS*	2.13	type I restriction-modification system, S subunit
105	PST1481		2.07	conserved hypothetical protein
106	PST1495		2.56	TonB-dependent siderophore receptor
107	PST1503		2.87	conserved hypothetical protein
108	PST1513		2.01	conserved hypothetical protein
109	PST1520		2.3	conserved hypothetical protein
110	PST1521		3.14	outer membrane protein
111	PST1561		2.12	ribosomal subunit interface protein, putative
112	PST1563	*adhC*	2.07	alcohol dehydrogenase class III
113	PST1585		2.09	conserved hypothetical protein
114	PST1610		2.12	flavin monoamine oxidase-related protein

115	PST1633		2.2	DNA topoisomerase I: Restriction endonuclease
116	PST1641		2.21	putative membrane protein
117	PST1642		2.48	rhodanese domain protein
118	PST1643		3.81	conserved hypothetical protein
119	PST1644		2.49	conserved hypothetical protein
120	PST1673	*catC*	2.06	muconolactone isomerase
121	PST1675	*benE*	2.03	benzoate transport protein
122	PST1711	*exbD1*	2.53	TonB system transport protein
123	PST1712	*exbB1*	2.79	TonB system transport protein ExbB
124	PST1713		17.54	TonB protein, C-terminal domain
125	PST1714		2.35	predicted Zn-dependent protease
126	PST1715		4.03	TldD/PmbA family protein
127	PST1716	*mvaB*	2.04	hydroxymethylglutaryl-CoA lyase
128	PST1721		2.03	conserved hypothetical protein
129	PST1733		2.47	cell division inhibitor-related protein
130	PST1753		2.56	glycosyl transferase, group 1 family protein
131	PST1760		2.05	glycosyl transferase, group 2 family protein
132	PST1762		2.1	aminotransferase, DegT/DnrJ/EryC1/StrS family
133	PST1889		2.21	methyl-accepting chemotaxis protein
134	PST1912		2.46	conserved hypothetical protein
135	PST1920		2.06	conserved hypothetical protein
136	PST1941		2.02	transcriptional regulator, LysR family
137	PST1954		4.46	nitrite reductase [NAD(P)H], small subunit
138	PST1955		19.98	nitrite reductase [NAD(P)H] large subunit
139	PST1992		3.39	ABC transporter, periplasmic substrate-binding protein
140	PST1993		4.52	glutamate-ammonia ligase
141	PST1998		2.12	chain F, crystal structure of creatinine amidohydrolase (Creatininase)
142	PST2002		2.45	conserved hypothetical protein
143	PST2003		4.15	ABC-type nitrate/sulfonate/bicarbonate transport systems, periplasmic component
144	PST2009		2.24	conserved hypothetical protein
145	PST2011		2.51	ABC transporter, permease protein
146	PST2048		2	universal stress protein family
147	PST2137	*glgA*	2.23	glycogen synthase
148	PST2154		2.83	alpha-amylase family protein
149	PST2179	*strU*	2.46	NAD(P)-dependent oxidoreductase
150	PST2192	*mtlG*	2.08	mannitol ABC transporter, permease protein
151	PST2193	*mtlK*	2.02	maltose/maltodextrin ABC transporter, ATP-binding protein
152	PST2199		2.03	putative ABC transporter periplasmic solute-binding protein
153	PST2234		3.53	heat shock protein, HSP20 family
154	PST2335		2.03	aminotransferase

155	PST2373		2.16	conserved hypothetical protein
156	PST2381		4.09	conserved hypothetical protein
157	PST2400	*nasS*	2.4	nitrate-binding protein NasS
158	PST2402		6.53	conserved hypothetical protein
159	PST2406	*nasA*	7.17	nitrate transporter
160	PST2409		11.62	assimilatory nitrite reductase large subunit
161	PST2410		4.37	assimilatory nitrite reductase small subunit
162	PST2411		2.4	assimilatory nitrate reductase
163	PST2425		2.05	conserved hypothetical protein
164	PST2496		2.79	conserved hypothetical protein
165	PST2498		2.43	conserved hypothetical protein
166	PST2499		2.46	conserved hypothetical protein
167	PST2500		2.12	N-acetyl-mannosamine transferase
168	PST2501		2.61	capsular polysaccharide biosynthesis protein
169	PST2508		4.99	methyl-accepting chemotaxis transducer
170	PST2575	*fliR*	2.2	flagellar biosynthetic protein FliR
171	PST2746		2.03	conserved hypothetical protein
172	PST2747		6.57	chromosome segregation ATPase
173	PST2748		3.83	OmpA family protein
174	PST2764		2.28	conserved hypothetical protein
175	PST2837		2.22	conserved hypothetical protein
176	PST2862		7.27	nucleoside-binding outer membrane protein
177	PST2885		2.15	circadian oscillation regulator
178	PST2897		2.55	probable oxidoreductase
179	PST2899		4.53	hypothetical protein
180	PST2900		3.59	probable ABC transporter, ATP-binding component
181	PST2906		4.03	conserved hypothetical protein
182	PST2907		3.56	ABC transporter, ATP-binding protein
183	PST2913	*topB*	5	DNA topoisomerase III
184	PST2922		2.59	hydrolase, alpha/beta fold family
185	PST2982	*braC*	2.02	branched-chain amino acid transport protein BraC
186	PST3079		11.08	membrane protein, bmp family
187	PST3080		3.92	oxidoreductase, 2OG-Fe(II) oxygenase family
188	PST3099		3.97	nucleoside-binding outer membrane protein
189	PST3101		2.71	transcriptional regulator, TetR family
190	PST3106		3.24	NAD-dependent aldehyde dehydrogenase
191	PST3127		2.4	esterase EstA
192	PST3129		25.51	conserved hypothetical protein
193	PST3130	*iciA*	2.03	chromosome initiation inhibitor
194	PST3159		2.08	prolyl oligopeptidase family protein
195	PST3160		2.1	biotin carboxylase

196	PST3170		2.45	conserved hypothetical protein
197	PST3237		2.39	conserved hypothetical protein
198	PST3246		4	PqiB family protein
199	PST3247		2.33	paraquat-inducible protein A
200	PST3253		2.05	membrane protein, putative
201	PST3305		4.04	osmY-related protein
202	PST3319	*ftsH*	2.06	cell division protein FtsH
203	PST3341		4.37	conserved hypothetical protein
204	PST3361	*czcA*	3.64	heavy metal efflux pump CzcA
205	PST3371		2.22	conserved hypothetical protein
206	PST3408		2.88	permease, drug/metabolite transporter (DMT) superfamily
207	PST3414		2.26	conserved hypothetical protein
208	PST3416		2.26	Co/Zn/Cd efflux system component
209	PST3417		2.87	predicted transcriptional regulators
210	PST3422		2.07	out membrane porin
211	PST3428		2.35	cation efflux system protein
212	PST3438		2.11	conserved hypothetical protein
213	PST3454		2.44	conserved hypothetical protein
214	PST3455		2.05	ATPase of the AAA+ class
215	PST3503		2.56	probable periplasmic protein
216	PST3510	*dnrS*	2.48	transcriptional regulator DnrS
217	PST3521	*norB*	2.24	nitric-oxide reductase subunit B
218	PST3522	*norC*	3.01	nitric-oxide reductase subunit C
219	PST3532	*nirS*	3.17	cytochrome cd1 nitrite reductase precursor
220	PST3533	*nirQ*	2.68	denitrification regulatory protein nirQ
221	PST3536	*nirJ*	2.8	heme d1 biosynthesis protein NirJ
222	PST3566	*cynS*	4.15	cyanate lyase
223	PST3569	*codB*	2.4	cytosine transporter
224	PST3570	*codA*	2.7	cytosine deaminase
225	PST3584		2.01	oxidoreductase, 2OG-Fe(II) oxygenase family
226	PST3597		10.74	bacterial luciferase family protein
227	PST3598		7.91	isochorismatase family protein
228	PST3621		3.15	transcriptional regulator, AraC family
229	PST3680		3.55	ethanolamine transporter
230	PST3681	*eutB*	2.1	ethanolamine ammonia-lyase large subunit
231	PST3720		12.5	branched-chain amino acid ABC transporter, periplasmic amino acid-binding protein, putative
232	PST3726	*ureD-2*	4.04	urease accessory protein UreD
233	PST3727	*ureA*	4.39	urease, gamma subunit
234	PST3736	*ureE*	4.51	urease accessory protein UreE
235	PST3737	*ureF-2*	2.5	urease accessory protein UreF

236	PST3738	*ureG*	2.06	urease accessory protein UreG
237	PST3780	*rodA*	2.79	rod-shape-determining protein RodA
238	PST3781	*mrdA-2*	2.01	penicillin-binding protein 2
239	PST3795		2.84	ribosome-associated GTPase
240	PST3909		3.62	conserved hypothetical protein
241	PST3910		4	conserved hypothetical protein
242	PST3912		6.46	hypothetical protein
243	PST3913		5.61	conserved hypothetical protein
244	PST3929		2.18	ABC transporter, ATP-binding protein
245	PST3934		2.43	conserved hypothetical protein
246	PST3971	*metW*	2.41	methionine biosynthesis protein MetW
247	PST4051		3.82	conserved hypothetical protein
248	PST4084		2.3	ABC-type amino acid transport/signal transduction systems, periplasmic component/domain
249	PST4091	*nasR*	2.27	nitrate-and nitrite-responsive positive regulator
250	PST4092	*nasF*	20.2	NrtA-type periplasmic nitrate transport binding protein, probable
251	PST4093	*nasE*	5.13	nitrate ABC transporter permease protein
252	PST4094	*nasD*	22.71	nitrate ABC transporter, ATP-binding protein, putative
253	PST4095		4.1	putative acetyltransferase
254	PST4124		2.48	periplasmic binding protein, putative
255	PST4164	*coxA*	2.37	cytochrome c oxidase, subunit I
(b) Downregulated genes				
1	PST0002	*dnaN*	0.49	DNA polymerase III, beta subunit
2	PST0107	*rpmB*	0.06	50S ribosomal protein L28
3	PST0186	*accC-2*	0.4	acetyl-CoA carboxylase, biotin carboxylase
4	PST0187	*oadA*	0.25	oxaloacetate decarboxylase, alpha subunit
5	PST0189		0.27	conserved hypothetical protein
6	PST0191		0.32	carbonic anhydrase
7	PST0335	*tatA*	0.31	translocation protein TatA
8	PST0355	*typA*	0.16	GTP-binding protein TypA
9	PST0389	*phoP*	0.5	two-component response regulator PhoP
10	PST0390	*oprH*	0.31	outer membrane protein H1 precursor
11	PST0398	*serA*	0.37	D-3-phosphoglycerate dehydrogenase
12	PST0400		0.48	fumarylacetoacetate hydrolase family protein
13	PST0460	*rpoZ*	0.49	RNA polymerase omega subunit
14	PST0463	*rph*	0.43	ribonuclease PH
15	PST0483	*dadA*	0.49	D-amino acid dehydrogenase, small subunit
16	PST0485	*cycB*	0.47	cytochrome c5
17	PST0543	*rho*	0.42	transcription termination factor Rho
18	PST0561	*gltB*	0.1	glutamate synthase large chain precursor
19	PST0562	*gltD*	0.12	glutamate synthase, small subunit

20	PST0678		0.49	lipoprotein, putative
21	PST0714	*rpsU*	0.21	ribosomal protein S21
22	PST0728	*surA*	0.37	peptidyl-prolyl cis-trans isomerase SurA
23	PST0773	*rplK*	0.33	50S ribosomal protein L11
24	PST0774	*rplA*	0.13	ribosomal protein L1
25	PST0775	*rplJ*	0.21	50S ribosomal protein L10
26	PST0776	*rplL*	0.26	50S ribosomal protein L7 / L12
27	PST0777	*rpoB*	0.13	DNA-directed RNA polymerase beta chain
28	PST0779	*rpsL*	0.17	30S ribosomal protein S12
29	PST0780	*rpsG*	0.33	ribosomal protein S7
30	PST0782	*tuf-1*	0.13	translation elongation factor Tu
31	PST0784	*rplC*	0.33	ribosomal protein L3
32	PST0785	*rplD*	0.27	ribosomal protein L4
33	PST0786	*rplW*	0.12	50S ribosomal protein L23
34	PST0788	*rpsS*	0.05	ribosomal protein S19
35	PST0789	*rplV*	0.07	ribosomal protein L22
36	PST0791	*rplP*	0.21	ribosomal protein L16
37	PST0792	*rpmC*	0.04	50S ribosomal protein L29
38	PST0793	*rpsQ*	0.1	ribosomal protein S17
39	PST0794	*rplN*	0.08	ribosomal protein L14
40	PST0795	*rplX*	0.05	ribosomal protein L24
41	PST0796	*rplE*	0.21	50S ribosomal protein L5
42	PST0797	*rpsN*	0.19	30S ribosomal protein S14
43	PST0798	*rpsH*	0.16	ribosomal protein S8
44	PST0799	*rplF*	0.23	50S ribosomal protein L6
45	PST0800	*rplR*	0.04	50S ribosomal protein L18
46	PST0801	*rpsE*	0.24	ribosomal protein S5
47	PST0803	*rplO*	0.22	50S ribosomal protein L15
48	PST0804	*secY*	0.44	secretion protein SecY
49	PST0806	*rpsM*	0.11	ribosomal protein S13
50	PST0807	*rpsK*	0.12	30S ribosomal protein S11
51	PST0808	*rpsD*	0.07	ribosomal protein S4
52	PST0809	*rpoA*	0.18	DNA-directed RNA polymerase alpha chain
53	PST0817	*oprG*	0.34	outer membrane protein OprG
54	PST0838	*ribH*	0.38	riboflavin synthase, beta subunit
55	PST0846		0.42	TonB-dependent receptor, B12 family
56	PST0852		0.14	predicted membrane protein
57	PST0854		0.22	conserved hypothetical protein
58	PST0895	*narM*	0.47	nitrate transporter
59	PST0899	*narI*	0.48	respiratory nitrate reductase gamma chain
60	PST0904		0.44	cytochrome b561

61	PST0913	*colR*	0.32	DNA-binding response regulator ColR
62	PST0916		0.36	lipopolysaccharide kinase
63	PST0917		0.32	diacylglycerol kinase
64	PST0918		0.21	membrane protein, putative
65	PST0934	*gdhA*	0.45	glutamate dehydrogenase
66	PST0940	*glyA3*	0.23	serine hydroxymethyltransferase
67	PST0955	*ispB*	0.28	octylprenyl diphosphate synthase
68	PST0956	*rplU*	0.14	ribosomal protein L21
69	PST0957	*rpmA*	0.1	ribosomal protein L27
70	PST0958	*obg*	0.21	GTP-binding protein Obg
71	PST0959	*proB*	0.47	glutamate 5-kinase
72	PST0960	*creA*	0.42	CreA family protein
73	PST0961	*rpsT*	0.2	30S ribosomal protein S20
74	PST0963	*ribF*	0.46	riboflavin kinase/FAD synthase
75	PST0965	*lspA*	0.36	lipoprotein signal peptidase
76	PST0966	*fkpB*	0.47	peptidyl-prolyl cis-trans isomerase, FKBP-type
77	PST0994		0.42	probable ornithine decarboxylase
78	PST1015	*mreB*	0.38	rod shape-determining protein MreB
79	PST1035		0.37	phosphatase, YrbI family
80	PST1039	*ttg2C*	0.49	toluene tolerance ABC transporter, periplasmic substrate-binding protein
81	PST1043	*ttg2F*	0.34	toluene-tolerance protein
82	PST1044	*murA*	0.25	UDP-N-acetylglucosamine 1-carboxyvinyltransferase
83	PST1045	*hisG*	0.49	ATP-phosphoribosyltransferase
84	PST1050	*cysD*	0.43	ATP sulfurylase small subunit
85	PST1051	*cysN*	0.41	ATP sulfurylase GTP-binding subunit/APS kinase
86	PST1061	*rplM*	0.29	50S ribosomal protein L13
87	PST1062	*rpsI*	0.18	ribosomal protein S9
88	PST1063	*petA*	0.36	ubiquinol--cytochrome c reductase, iron-sulfur subunit
89	PST1064	*petB*	0.43	ubiquinol--cytochrome c reductase, cytochrome b
90	PST1087	*ftsZ*	0.23	cell division protein FtsZ
91	PST1088	*lpxC*	0.25	UDP-3-O-acyl-N-acetylglucosamine deacetylase
92	PST1101		0.3	conserved hypothetical protein
93	PST1102	*mqoB*	0.47	malate:quinone oxidoreductase
94	PST1128	*slyD*	0.45	peptidyl-prolyl cis-trans isomerase, FKBP-type
95	PST1129		0.44	glutathione peroxidase
96	PST1134		0.44	conserved hypothetical protein
97	PST1138		0.4	flavodoxin
98	PST1147		0.32	probable fumarase
99	PST1165	*purU1*	0.37	formyltetrahydrofolate deformylase
100	PST1167		0.46	hypothetical protein
101	PST1185		0.48	anhydrase, family 3 protein

102	PST1190	*ffh*	0.35	signal recognition particle protein Ffh
103	PST1191	*rpsP*	0.12	ribosomal protein S16
104	PST1192	*rimM*	0.23	16S rRNA processing protein
105	PST1193	*trmD*	0.16	tRNA (guanine-N1)-methyltransferase
106	PST1194	*rplS*	0.15	50S ribosomal protein L19
107	PST1199	*thrC*	0.45	threonine synthase
108	PST1203	*argG*	0.43	argininosuccinate synthase
109	PST1210		0.49	electron transport complex protein rnfC
110	PST1220		0.45	conserved hypothetical protein
111	PST1227	*mucD*	0.39	serine protease MucD precursor
112	PST1228	*lepA*	0.5	GTP-binding protein LepA
113	PST1241		0.26	probable transcriptional regulator
114	PST1281	*cydA1*	0.45	cytochrome d ubiquinol oxidase, subunit I
115	PST1282	*cydB1*	0.26	cytochrome d terminal oxidase polypeptide subunit II
116	PST1289		0.3	TonB-dependent receptor
117	PST1290		0.19	conserved hypothetical protein
118	PST1291	*cobO*	0.27	cob(I)alamin adenosyltransferase
119	PST1292	*cobB*	0.42	cobyrinic acid a,c-diamide synthase
120	PST1369	*alaS*	0.39	alanyl-tRNA synthetase
121	PST1377		0.36	oxaloacetate decarboxylase, alpha subunit
122	PST1410		0.44	conserved hypothetical protein
123	PST1423	*minE*	0.38	cell division topological specificity factor MinE
124	PST1424	*minD*	0.32	cell division inhibitor MinD
125	PST1504	*fdxA*	0.37	ferredoxin I
126	PST1518	*fpr*	0.49	ferredoxin--NADP reductase
127	PST1537	*rpsB*	0.18	30S ribosomal protein S2
128	PST1538	*tsf*	0.31	elongation factor Ts
129	PST1545		0.39	outer membrane protein, bacterial surface antigen family
130	PST1553	*accA*	0.5	acetyl-CoA carboxylase, carboxyl transferase, alpha subunit
131	PST1566	*ispF*	0.43	2C-methyl-D-erythritol 2,4-cyclodiphosphate
132	PST1598		0.35	peptidyl-prolyl cis-trans isomerase C
133	PST1637	*nrdA*	0.36	ribonucleoside reductase, large chain
134	PST1656	*vacJ*	0.44	VacJ-like lipoprotein
135	PST1657		0.46	conserved hypothetical protein
136	PST1729	*topA*	0.46	DNA topoisomerase I
137	PST1775	*asd-1*	0.31	aspartate-semialdehyde dehydrogenase
138	PST1784	*purF*	0.38	amidophosphoribosyltransferase
139	PST1785	*metZ*	0.37	O-succinylhomoserine sulfhydrylase
140	PST1821		0.28	conserved hypothetical protein
141	PST1837		0.26	cytochrome c oxidase, cbb3-type, subunit III
142	PST1838	*ccoQ-2*	0.17	cytochrome c oxidase, cbb3-type, CcoQ subunit

143	PST1840	*ccoN-2*	0.27	cytochrome c oxidase, cbb3-type, subunit I
144	PST1841		0.17	cytochrome c oxidase, cbb3-type, subunit III
145	PST1842		0.14	cytochrome c oxidase, cbb3-type, subunit II
146	PST1843	*ccoN-1*	0.4	cytochrome c oxidase, cbb3-type, subunit I
147	PST1870	*gltA*	0.46	citrate synthase
148	PST1873	*sdhA*	0.38	succinate dehydrogenase (A subunit)
149	PST1874	*sdhB*	0.33	succinate dehydrogenase, iron-sulfur protein
150	PST1875	*sucA*	0.32	2-oxoglutarate dehydrogenase, E1 component
151	PST1876	*sucB*	0.2	dihydrolipoamide succinyltransferase (E2 subunit)
152	PST1877	*lpdG*	0.33	lipoamide dehydrogenase-glc
153	PST1878	*sucC*	0.27	succinyl-CoA synthetase beta chain
154	PST1903	*selD*	0.47	selenide, water dikinase
155	PST2022	*oprI*	0.17	outer membrane lipoprotein OprI
156	PST2028	*thrH*	0.42	homoserine kinase
157	PST2039	*ppsA*	0.49	phosphoenolpyruvate synthase
158	PST2040	*menG*	0.48	s-adenosylmethionine:2-demethylmenaquinone methyltransferase
159	PST2046	*acnB*	0.42	aconitate hydratase 2
160	PST2054	*folD*	0.46	5,10-methylene-tetrahydrofolate dehydrogenase / cyclohydrolase
161	PST2092		0.42	redox-active disulfide protein
162	PST2223	*metH*	0.29	methionine synthase
163	PST2236	*cysI*	0.36	sulfite reductase
164	PST2237		0.47	conserved hypothetical protein
165	PST2297	*infA*	0.2	translation initiation factor IF-1
166	PST2302	*idh*	0.35	isocitrate dehydrogenase
167	PST2306	*purB*	0.37	adenylosuccinate lyase
168	PST2307		0.36	conserved hypothetical protein
169	PST2308		0.35	probable acetyl transferase
170	PST2325		0.35	oxidoreductase, short chain dehydrogenase/reductase family
171	PST2326		0.43	pseudouridine synthase
172	PST2333	*folE2*	0.4	GTP cyclohydrolase I precursor
173	PST2337	*rpsA*	0.25	30S ribosomal protein S1
174	PST2338	*cmk*	0.32	cytidylate kinase
175	PST2363	*ihfA*	0.43	integration host factor, alpha subunit
176	PST2364	*pheT*	0.46	phenylalanyl-tRNA synthetase, beta subunit
177	PST2366	*rplT*	0.26	50S ribosomal protein L20
178	PST2367	*rpmI*	0.2	ribosomal protein L35
179	PST2385		0.45	conserved hypothetical protein
180	PST2396		0.24	conserved hypothetical protein
181	PST2507		0.25	ATP-dependent RNA helicase, DEAD box family
182	PST2512	*efp*	0.29	translation elongation factor P
183	PST2551	*ccmE*	0.45	cytochrome C-type biogenesis protein CcmE

184	PST2552	*ccmD*	0.45	cytochrome c-type biogenesis protein CcmD
185	PST2565	*cheB*	0.46	protein-glutamate methylesterase CheB
186	PST2566	*cheA*	0.32	chemotaxis histidine kinase CheA
187	PST2567	*cheZ*	0.43	chemotaxis protein CheZ
188	PST2568	*cheY*	0.41	two-component response regulator CheY
189	PST2581	*fliL*	0.4	flagellar protein FliL
190	PST2591	*fliE*	0.43	flagellar hook-basal body complex protein FliE
191	PST2605	*etfB*	0.44	electron transfer flavoprotein beta-subunit
192	PST2606		0.32	conserved hypothetical protein
193	PST2607		0.47	electron transfer flavoprotein-ubiquinone oxidoreductase
194	PST2620	*fabF*	0.48	3-oxoacyl-(acyl-carrier-protein) synthase II
195	PST2621	*acpP*	0.2	acyl carrier protein
196	PST2622		0.36	3-oxoacyl-(acyl-carrier-protein) reductase
197	PST2623		0.28	malonyl-CoA-[acyl-carrier-protein] transacylase
198	PST2625	*rpmF*	0.19	50S ribosomal protein L32
199	PST2626		0.23	predicted metal-binding, possibly nucleic acid-binding protein
200	PST2652	*nqrD*	0.41	Na+-translocating NADH:uniquinone oxidoreductase subunit Nqr4
201	PST2653	*nqrC*	0.29	Na+-translocating NADH:ubiquinone oxidoreductase subunit Nrq3
202	PST2654	*nqrB*	0.29	Na+-translocating NADH:ubiquinone oxidoreductase subunit Nrq2
203	PST2655	*nqrA*	0.39	Na+-translocating NADH:ubiquinone oxidoreductase subunit Nrq1
204	PST2788		0.46	phosphoribosylaminoimidazole-succinocarboxamidesynthase
205	PST2789		0.39	lipoprotein, putative
206	PST2796	*nadA*	0.45	quinolinate synthetase A
207	PST2797		0.4	acetyl-CoA hydrolase
208	PST2801		0.4	conserved hypothetical protein
209	PST2802	*oprL*	0.41	peptidoglycan-associated lipoprotein OprL
210	PST2805	*tolR*	0.32	TolR protein
211	PST2820		0.44	lipoprotein, putative
212	PST2830	*purM*	0.43	phosphoribosylformylglycinamidine cyclo-ligase
213	PST2849	*glpD-1*	0.34	glycerol-3-phosphate dehydrogenase
214	PST2850	*metE*	0.04	5-methyltetrahydropteroyltriglutamate- homocysteine S-methyltransferase
215	PST2977		0.34	phosphonate ABC transporter, periplasmic phosphonate-binding protein, putative
216	PST3007	*guaA*	0.31	GMP synthase
217	PST3008	*guaB*	0.42	inosine-5'-monophosphate dehydrogenase
218	PST3014	*oprC*	0.28	outer membrane protein OprC
219	PST3028		0.44	PQQ enzyme repeat domain protein
220	PST3031	*ispG*	0.46	1-hydroxy-2-methyl-2-(E)-butenyl 4-diphosphate synthase
221	PST3032		0.47	transcriptional regulator, Cro/CI family
222	PST3034		0.46	radical SAM enzyme, Cfr family
223	PST3035	*ndk*	0.49	nucleoside diphosphate kinase

224	PST3037	*fdx2*	0.48	ferredoxin
225	PST3041	*iscU*	0.38	iron-binding protein IscU
226	PST3045		0.45	probable methyltransferase
227	PST3046		0.39	inositol-1-monophosphatase
228	PST3049	*secD*	0.41	secretion protein SecD
229	PST3050	*yajC*	0.47	preprotein translocase, YajC subunit
230	PST3052	*queA*	0.47	S-adenosylmethionine:trna ribosyltransferase-isomerase
231	PST3145	*groEL*	0.49	GroEL protein
232	PST3165	*argJ*	0.39	glutamate N-acetyltransferase/amino-acid acetyltransferase
233	PST3189	*prsA*	0.23	ribose-phosphate pyrophosphokinase
234	PST3192	*ychF*	0.3	GTP-binding protein YchF
235	PST3273	*accB*	0.37	biotin carboxyl carrier protein (BCCP)
236	PST3277		0.44	TIM-barrel protein, putative, NifR3 family
237	PST3278	*fis*	0.2	DNA-binding protein Fis
238	PST3279	*purH*	0.19	phosphoribosylaminoimidazolecarboxamideformyltransferase
239	PST3280	*purD*	0.31	phosphoribosylamine--glycine ligase
240	PST3298		0.28	aspartate 1-decarboxylase precursor
241	PST3306	*pnp*	0.35	polyribonucleotide nucleotidyltransferase
242	PST3307	*rpsO*	0.38	30S ribosomal protein S15
243	PST3309	*rbfA*	0.3	ribosome-binding factor A
244	PST3310	*infB*	0.28	translation initiation factor IF-2
245	PST3311	*nusA*	0.19	N utilization substance protein A
246	PST3312		0.48	conserved hypothetical protein
247	PST3315	*secG*	0.33	preprotein translocase, SecG subunit
248	PST3322	*greA*	0.4	transcription elongation factor GreA
249	PST3324	*carA*	0.35	carbamoyl-phosphate synthase small chain
250	PST3337		0.42	Fe-S oxidoreductase
251	PST3573		0.31	cytosolic long-chain acyl-CoA thioester hydrolase family protein
252	PST3629		0.15	ATPase, putative
253	PST3653	*rplI*	0.16	ribosomal protein L9
254	PST3654		0.07	membrane protein, putative
255	PST3655	*rpsR*	0.26	30S ribosomal protein S18
256	PST3656	*rpsF*	0.11	ribosomal protein S6
257	PST3663	*purA*	0.44	adenylosuccinate synthetase
258	PST3664		0.38	ATP phosphoribosyltransferase regulatory subunit, putative
259	PST3667	*hflX*	0.49	GTP-binding protein HflX
260	PST3686	*ppa-1*	0.27	inorganic pyrophosphatase
261	PST3696		0.49	thiol-disulfide isomerase and thioredoxins
262	PST3771		0.44	conserved hypothetical protein
263	PST3774	*lipA*	0.43	lipoate synthase
264	PST3783		0.43	iojap-related protein

265	PST3827		0.46	uncharacterized protein
266	PST3850	*ilvE*	0.26	branched-chain amino acid transferase
267	PST3853	*aceF*	0.41	dihydrolipoamide acetyltransferase
268	PST3920	*tktA*	0.48	transketolase
269	PST3926	*metK*	0.22	S-adenosylmethionine synthetase
270	PST3935	*sahH*	0.15	S-adenosyl-L-homocysteine hydrolase
271	PST3936	*metF*	0.27	5,10-methylenetetrahydrofolate reductase
272	PST4002	*coaD*	0.48	pantetheine-phosphate adenylyltransferase
273	PST4059	*dsbA*	0.39	thiol:disulfide interchange protein DsbA
274	PST4060		0.16	cytochrome c4 precursor
275	PST4063	*gcvH1*	0.49	glycine cleavage system protein H1
276	PST4064	*gcvT1*	0.35	glycine-cleavage system protein T1
277	PST4077	*secB*	0.32	protein-export protein SecB
278	PST4088	*hisA*	0.41	phosphoribosylformimino-5-aminoimidazole carboxamide
279	PST4089		0.42	conserved hypothetical protein
280	PST4108		0.44	conserved hypothetical protein
281	PST4110		0.47	D-amino acid dehydrogenase, small subunit
282	PST4190	*atpC*	0.28	ATP synthase F1, epsilon subunit
283	PST4191	*atpD*	0.16	ATP synthase beta chain
284	PST4192	*atpG*	0.16	ATP synthase gamma chain
285	PST4193	*atpA*	0.08	ATP synthase F1, alpha subunit
286	PST4194	*atpH*	0.11	ATP synthase delta chain
287	PST4195	*atpF*	0.05	ATP synthase B chain
288	PST4196	*atpE*	0.07	ATP synthase F0, C subunit
289	PST4197	*atpB*	0.15	ATP synthase F0, A subunit
290	PST4198	*atpI*	0.27	ATP synthase protein I
291	PST4201	*gidB*	0.41	glucose inhibited division protein B
292	PST4211	*yidC*	0.42	inner membrane protein, 60 kDa
293	PST4212		0.17	conserved hypothetical protein
294	PST4213	*rnpA*	0.12	ribonuclease P protein component
