# Supplementary material for: Global transcriptional analysis of nitrogen fixation and ammonium repression in root-associated Pseudomonas stutzeri A1501
Source: BMC Genomics. 2010 Jan 7;11:11. doi: 10.1186/1471-2164-11-11 (PMC2820453; doi:10.1186/1471-2164-11-11)
Supplement: Additional file 2 — Upregulation (a) or downregulation (b) of genes 10 minutes after exposure of bacteria to ammonium shock. [file 1471-2164-11-11-S2.PDF]

Additional file 2

Upregulation (a) or downregulation (b) of genes 10 minutes after exposure of bacteria to ammonium shock

Number	Gene ID	Gene name	Ammonia shock	Nitrogen fixation[a]	Functional description
**(a) Upregulated genes**					
1	PST0107	*rpmB*	10.15	Down	50S ribosomal protein L28
2	PST0134		2.17	NR	probable signal transduction protein
3	PST0186	*accC-2*	2.61	Down	acetyl-CoA carboxylase, biotin carboxylase
4	PST0187	*oadA*	2.58	Down	oxaloacetate decarboxylase, alpha subunit
5	PST0189		2.40	Down	conserved hypothetical protein
6	PST0191		2.04	Down	carbonic anhydrase
7	PST0211	*cysP*	3.62	NR	sulfate ABC transporter, periplasmic sulfate-binding protein
8	PST0213		2.82	NR	conserved hypothetical protein
9	PST0214	*lsfA*	2.85	NR	antioxidant protein LsfA
10	PST0218		2.27	NR	alkyl sulfatase
11	PST0335	*tatA*	3.11	Down	translocation protein TatA
12	PST0355	*typA*	4.55	Down	GTP-binding protein TypA
13	PST0390	*oprH*	2.08	Down	outer membrane protein H1 precursor
14	PST0395		2.05	NR	oligopeptide transporter, OPT family
15	PST0398	*serA*	2.97	Down	D-3-phosphoglycerate dehydrogenase
16	PST0460	*rpoZ*	2.80	Down	RNA polymerase omega subunit
17	PST0469	*argB*	2.12	NR	acetylglutamate kinase
18	PST0543	*rho*	2.67	Down	transcription termination factor Rho
19	PST0561	*gltB*	9.46	Down	glutamate synthase large chain precursor
20	PST0562	*gltD*	5.71	Down	glutamate synthase, small subunit
21	PST0569		2.12	NR	transporter sodium/sulfate symporter family
22	PST0678		2.06	Down	lipoprotein, putative
23	PST0682		2.12	NR	membrane fusion protein
24	PST0714	*rpsU*	3.70	Down	ribosomal protein S21
25	PST0728	*surA*	2.19	Down	peptidyl-prolyl cis-trans isomerase SurA
26	PST0773	*rplK*	2.33	Down	50S ribosomal protein L11
27	PST0774	*rplA*	4.17	Down	ribosomal protein L1
28	PST0775	*rplJ*	3.75	Down	50S ribosomal protein L10
29	PST0776	*rplL*	2.04	Down	50S ribosomal protein L7 / L12
30	PST0777	*rpoB*	5.86	Down	DNA-directed RNA polymerase beta chain
31	PST0779	*rpsL*	4.80	Down	30S ribosomal protein S12
32	PST0780	*rpsG*	3.44	Down	ribosomal protein S7
33	PST0782	*tuf-1*	3.47	Down	translation elongation factor Tu

34	PST0784	*rplC*	2.71	Down	ribosomal protein L3
35	PST0785	*rplD*	3.34	Down	ribosomal protein L4
36	PST0786	*rplW*	4.92	Down	50S ribosomal protein L23
37	PST0788	*rpsS*	9.28	Down	ribosomal protein S19
38	PST0789	*rplV*	6.79	Down	ribosomal protein L22
39	PST0791	*rplP*	2.88	Down	ribosomal protein L16
40	PST0792	*rpmC*	11.57	Down	50S ribosomal protein L29
41	PST0793	*rpsQ*	7.24	Down	ribosomal protein S17
42	PST0794	*rplN*	5.16	Down	ribosomal protein L14
43	PST0795	*rplX*	9.16	Down	ribosomal protein L24
44	PST0796	*rplE*	3.73	Down	50S ribosomal protein L5
45	PST0797	*rpsN*	3.09	Down	30S ribosomal protein S14
46	PST0798	*rpsH*	4.61	Down	ribosomal protein S8
47	PST0799	*rplF*	4.04	Down	50S ribosomal protein L6
48	PST0800	*rplR*	8.67	Down	50S ribosomal protein L18
49	PST0801	*rpsE*	3.56	Down	ribosomal protein S5
50	PST0803	*rplO*	3.13	Down	50S ribosomal protein L15
51	PST0806	*rpsM*	6.61	Down	ribosomal protein S13
52	PST0807	*rpsK*	4.20	Down	30S ribosomal protein S11
53	PST0808	*rpsD*	11.54	Down	ribosomal protein S4
54	PST0809	*rpoA*	5.21	Down	DNA-directed RNA polymerase alpha chain
55	PST0849		2.84	NR	predicted transcriptional regulator
56	PST0912		2.09	NR	conserved hypothetical protein
57	PST0913	*colR*	2.36	Down	DNA-binding response regulator ColR
58	PST0915		2.01	NR	SAM-dependent methyltransferase
59	PST0916		2.60	Down	lipopolysaccharide kinase
60	PST0917		4.09	Down	diacylglycerol kinase
61	PST0918		2.53	Down	membrane protein, putative
62	PST0936		2.19	NR	conserved hypothetical protein
63	PST0940	*glyA3*	4.65	Down	serine hydroxymethyltransferase
64	PST0945		2.63	NR	conserved hypothetical protein
65	PST0955	*ispB*	2.62	Down	octylprenyl diphosphate synthase
66	PST0956	*rplU*	6.06	Down	ribosomal protein L21
67	PST0957	*rpmA*	8.91	Down	ribosomal protein L27
68	PST0961	*rpsT*	8.14	Down	30S ribosomal protein S20
69	PST0994		4.21	Down	probable ornithine decarboxylase
70	PST1014	*gatC*	2.63	NR	Glu-tRNA(Gln) amidotransferase subunit C
71	PST1015	*mreB*	2.54	Down	rod shape-determining protein MreB
72	PST1017	*mreD*	2.05	NR	rod shape-determining protein MreD
73	PST1035		2.21	Down	phosphatase, YrbI family
74	PST1043	*ttg2F*	2.80	Down	toluene-tolerance protein

75	PST1044	*murA*	2.15	Down	UDP-N-acetylglucosamine 1-carboxyvinyltransferase
76	PST1050	*cysD*	3.22	Down	ATP sulfurylase small subunit
77	PST1051	*cysN*	3.63	Down	ATP sulfurylase GTP-binding subunit/APS kinase
78	PST1061	*rplM*	3.05	Down	50S ribosomal protein L13
79	PST1062	*rpsI*	3.78	Down	ribosomal protein S9
80	PST1063	*petA*	2.49	Down	ubiquinol--cytochrome c reductase, iron-sulfur subunit
81	PST1088	*lpxC*	2.44	Down	UDP-3-O-acyl-N-acetylglucosamine deacetylase
82	PST1101		2.68	Down	conserved hypothetical protein
83	PST1134		3.10	Down	conserved hypothetical protein
84	PST1147		2.94	Down	probable fumarase
85	PST1165	*purU1*	2.10	Down	formyltetrahydrofolate deformylase
86	PST1191	*rpsP*	6.80	Down	ribosomal protein S16
87	PST1192	*rimM*	3.21	Down	16S rRNA processing protein
88	PST1194	*rplS*	4.50	Down	50S ribosomal protein L19
89	PST1199	*thrC*	2.39	Down	threonine synthase
90	PST1203	*argG*	2.77	Down	argininosuccinate synthase
91	PST1210		2.62	Down	electron transport complex protein rnfC
92	PST1227	*mucD*	2.02	Down	serine protease MucD precursor
93	PST1241		4.54	Down	probable transcriptional regulator
94	PST1262	*metG*	2.04	NR	methionyl-tRNA synthetase
95	PST1265	*dcd*	2.10	NR	deoxycytidine triphosphate deaminase
96	PST1289		3.98	Down	TonB-dependent receptor
97	PST1290		2.64	Down	conserved hypothetical protein
98	PST1369	*alaS*	2.21	Down	alanyl-tRNA synthetase
99	PST1377		2.13	Down	oxaloacetate decarboxylase, alpha subunit
100	PST1423	*minE*	2.35	Down	cell division topological specificity factor MinE
101	PST1424	*minD*	3.72	Down	cell division inhibitor MinD
102	PST1504	*fdxA*	2.70	Down	ferredoxin I
103	PST1518	*fpr*	4.00	Down	ferredoxin--NADP reductase
104	PST1537	*rpsB*	4.56	Down	30S ribosomal protein S2
105	PST1538	*tsf*	2.13	Down	elongation factor Ts
106	PST1598		2.39	Down	peptidyl-prolyl cis-trans isomerase C
107	PST1621	*gnl*	2.02	NR	gluconolactonase
108	PST1636	*nrdB*	2.82	NR	ribonucleoside reductase, small chain
109	PST1637	*nrdA*	3.93	Down	ribonucleoside reductase, large chain
110	PST1657		2.14	Down	conserved hypothetical protein
111	PST1727	*foaB*	2.61	NR	fatty-acid oxidation complex beta-subunit
112	PST1747		2.06	NR	putative FeS oxidoreductase
113	PST1775	*asd-1*	2.00	Down	aspartate-semialdehyde dehydrogenase
114	PST1785	*metZ*	2.27	Down	O-succinylhomoserine sulfhydrylase
115	PST1821		3.02	Down	conserved hypothetical protein

116	PST1837		2.94	Down	cytochrome c oxidase, cbb3-type, subunit III
117	PST1838	*ccoQ-2*	4.72	Down	cytochrome c oxidase, cbb3-type, CcoQ subunit
118	PST1841		2.29	Down	cytochrome c oxidase, cbb3-type, subunit III
119	PST1842		4.43	Down	cytochrome c oxidase, cbb3-type, subunit II
120	PST1870	*gltA*	2.45	Down	citrate synthase
121	PST1874	*sdhB*	2.32	Down	succinate dehydrogenase, iron-sulfur protein
122	PST1875	*sucA*	2.67	Down	2-oxoglutarate dehydrogenase, E1 component
123	PST1876	*sucB*	3.31	Down	dihydrolipoamide succinyltransferase (E2 subunit)
124	PST1877	*lpdG*	3.37	Down	lipoamide dehydrogenase-glc
125	PST1878	*sucC*	3.99	Down	succinyl-CoA synthetase beta chain
126	PST1901		2.03	NR	lipoprotein, putative
127	PST2022	*oprI*	2.88	Down	outer membrane lipoprotein OprI
128	PST2027	*cysH*	3.74	NR	3'-phosphoadenosine-5'-phosphosulfate reductase
129	PST2028	*thrH*	2.89	Down	homoserine kinase
130	PST2223	*metH*	2.87	Down	methionine synthase
131	PST2236	*cysI*	5.46	Down	sulfite reductase
132	PST2237		3.60	Down	conserved hypothetical protein
133	PST2294	*trxB1*	2.01	NR	thioredoxin reductase 1
134	PST2297	*infA*	3.76	Down	translation initiation factor IF-1
135	PST2302	*idh*	2.09	Down	isocitrate dehydrogenase
136	PST2306	*purB*	2.67	Down	adenylosuccinate lyase
137	PST2325		2.19	Down	oxidoreductase, short chain dehydrogenase/reductase family
138	PST2337	*rpsA*	3.33	Down	30S ribosomal protein S1
139	PST2338	*cmk*	2.06	Down	cytidylate kinase
140	PST2366	*rplT*	3.12	Down	50S ribosomal protein L20
141	PST2367	*rpmI*	2.42	Down	ribosomal protein L35
142	PST2396		2.94	Down	conserved hypothetical protein
143	PST2512	*efp*	2.58	Down	translation elongation factor P
144	PST2537		2.02	NR	probable aminotransferase
145	PST2566	*cheA*	2.19	Down	chemotaxis histidine kinase CheA
146	PST2567	*cheZ*	2.99	Down	chemotaxis protein CheZ
147	PST2605	*etfB*	2.03	Down	electron transfer flavoprotein beta-subunit
148	PST2606		2.68	Down	conserved hypothetical protein
149	PST2621	*acpP*	3.74	Down	acyl carrier protein
150	PST2623		2.89	Down	malonyl-CoA-[acyl-carrier-protein] transacylase
151	PST2625	*rpmF*	3.50	Down	50S ribosomal protein L32
152	PST2626		2.31	Down	predicted metal-binding, possibly nucleic acid-binding protein
153	PST2652	*nqrD*	2.06	Down	Na+-translocating NADH:uniquinone oxidoreductase subunit Nqr4
154	PST2653	*nqrC*	2.79	Down	Na+-translocating NADH:ubiquinone oxidoreductase subunit Nrq3

155	PST2654	*nqrB*	2.67	Down	Na+-translocating NADH:ubiquinone oxidoreductase subunit Nrq2
156	PST2655	*nqrA*	2.31	Down	Na+-translocating NADH:ubiquinone oxidoreductase subunit Nrq1
157	PST2744	*adk*	2.15	NR	adenylate kinase
158	PST2754	*lysS*	2.29	NR	lysyl-tRNA synthetase
159	PST2805	*tolR*	2.19	Down	TolR protein
160	PST2850	*metE*	15.74	Down	5-methyltetrahydropteroyltriglutamate- homocysteine S-methyltransferase
161	PST2895		3.59	NR	tryptophan synthase beta chain
162	PST2977		3.11	Down	phosphonate ABC transporter, periplasmic phosphonate-binding protein, putative
163	PST3007	*guaA*	2.24	Down	GMP synthase
164	PST3008	*guaB*	2.02	Down	inosine-5'-monophosphate dehydrogenase
165	PST3014	*oprC*	3.06	Down	outer membrane protein OprC
166	PST3041	*iscU*	2.10	Down	iron-binding protein IscU
167	PST3046		2.45	Down	inositol-1-monophosphatase
168	PST3131		2.05	NR	transporter, LysE family
169	PST3132	*sodB*	2.54	NR	superoxide dismutase
170	PST3137		2.07	NR	iron-regulated protein A, putative
171	PST3165	*argJ*	2.33	Down	glutamate N-acetyltransferase/amino-acid acetyltransferase
172	PST3189	*prsA*	4.08	Down	ribose-phosphate pyrophosphokinase
173	PST3192	*ychF*	2.21	Down	GTP-binding protein YchF
174	PST3273	*accB*	2.88	Down	biotin carboxyl carrier protein (BCCP)
175	PST3278	*fis*	4.54	Down	DNA-binding protein Fis
176	PST3279	*purH*	5.45	Down	phosphoribosylaminoimidazolecarboxamideformyltransferase
177	PST3280	*purD*	2.17	Down	phosphoribosylamine--glycine ligase
178	PST3298		4.09	Down	aspartate 1-decarboxylase precursor
179	PST3309	*rbfA*	2.26	Down	ribosome-binding factor A
180	PST3310	*infB*	2.14	Down	translation initiation factor IF-2
181	PST3311	*nusA*	2.97	Down	N utilization substance protein A
182	PST3312		2.23	Down	conserved hypothetical protein
183	PST3315	*secG*	2.24	Down	preprotein translocase, SecG subunit
184	PST3336		2.09	NR	L-lactate permease
185	PST3337		2.40	Down	Fe-S oxidoreductase
186	PST3504		2.67	NR	predicted periplasmic lipoprotein involved in iron transport
187	PST3513		2.19	NR	probable protease
188	PST3521	*norB*	2.13	Up	nitric-oxide reductase subunit B
189	PST3523	*nirH*	2.97	NR	nitrite reductase heme biosynthesis H protein
190	PST3525	*nirL*	2.11	NR	heme d1 biosynthesis protein NirL
191	PST3526	*nirD*	2.33	NR	transcriptional regulators

192	PST3527	*nirF*	2.38	NR	heme d1 biosynthesis protein NirF
193	PST3529	*nirM*	2.08	NR	cytochrome c-551 precursor
194	PST3530	*nirB*	7.03	NR	denitrification system component cytochrome c-552
195	PST3531	*nirT*	11.07	NR	tetraheme protein NirT precursor
196	PST3537	*nirE*	2.25	NR	uroporphyrinogen-III C-methyltransferase
197	PST3538	*nirN*	3.50	NR	probable c-type cytochrome
198	PST3542		2.31	NR	methyl-accepting chemotaxis protein
199	PST3543		3.73	NR	short-chain dehydrogenase/reductase
200	PST3546	*nosL*	4.32	NR	lipoprotein involved in nitrous oxide reduction
201	PST3548	*nosF*	2.10	NR	ABC-type multidrug transport system, ATPase component
202	PST3549	*nosD*	4.71	NR	nitrous oxidase accessory protein
203	PST3550	*nosZ*	6.02	NR	nitrous-oxide reductase precursor
204	PST3551	*nosR*	4.77	NR	regulatory protein NosR
205	PST3573		2.17	Down	cytosolic long-chain acyl-CoA thioester hydrolase family protein
206	PST3629		5.78	Down	ATPase, putative
207	PST3653	*rplI*	3.97	Down	ribosomal protein L9
208	PST3654		2.72	Down	membrane protein, putative
209	PST3655	*rpsR*	3.49	Down	30S ribosomal protein S18
210	PST3656	*rpsF*	6.47	Down	ribosomal protein S6
211	PST3686	*ppa-1*	3.93	Down	inorganic pyrophosphatase
212	PST3735		2.11	NR	histone acetyltransferase HPA2
213	PST3761	*miaB*	2.33	NR	tRNA-i(6)A37 thiotransferase enzyme MiaB
214	PST3774	*lipA*	2.74	Down	lipoate synthase
215	PST3827		2.02	Down	uncharacterized protein
216	PST3850	*ilvE*	3.73	Down	branched-chain amino acid transferase
217	PST3860		2.44	NR	probable acyl-CoA dehydrogenase
218	PST3866	*bioB*	2.39	NR	biotin synthase
219	PST3920	*tktA*	2.53	Down	transketolase
220	PST3926	*metK*	5.20	Down	S-adenosylmethionine synthetase
221	PST3935	*sahH*	7.55	Down	S-adenosyl-L-homocysteine hydrolase
222	PST3936	*metF*	4.85	Down	5,10-methylenetetrahydrofolate reductase
223	PST3950	*pilJ*	3.59	NR	twitching motility protein PilJ
224	PST4002	*coaD*	2.63	Down	pantetheine-phosphate adenylyltransferase
225	PST4059	*dsbA*	2.20	Down	thiol:disulfide interchange protein DsbA
226	PST4060		4.79	Down	cytochrome c4 precursor
227	PST4066		6.36	NR	iron ABC transporter, periplasmic iron-binding protein
228	PST4077	*secB*	2.33	Down	protein-export protein SecB
229	PST4089		2.04	Down	conserved hypothetical protein
230	PST4098		2.24	NR	Fe-S cluster protector protein
231	PST4191	*atpD*	3.27	Down	ATP synthase beta chain
232	PST4192	*atpG*	3.44	Down	ATP synthase gamma chain

233	PST4193	*atpA*	5.58	Down	ATP synthase F1, alpha subunit
234	PST4194	*atpH*	6.17	Down	ATP synthase delta chain
235	PST4195	*atpF*	16.12	Down	ATP synthase B chain
236	PST4196	*atpE*	5.96	Down	ATP synthase F0, C subunit
237	PST4197	*atpB*	3.78	Down	ATP synthase F0, A subunit
238	PST4198	*atpI*	2.14	Down	ATP synthase protein I
239	PST4201	*gidB*	2.62	Down	glucose inhibited division protein B
240	PST4202	*gidA*	2.22	NR	glucose-inhibited division protein A
241	PST4211	*yidC*	2.39	Down	inner membrane protein, 60 kDa
242	PST4212		5.07	Down	conserved hypothetical protein
243	PST4213	*rnpA*	7.74	Down	ribonuclease P protein component
**(b) Downregulated genes**					
1	PST0029		0.31	Up	conserved hypothetical protein
2	PST0035		0.39	Up	coserved hypothetical protein
3	PST0064		0.42	NR	probable glutamine amidotransferase
4	PST0200		0.35	Up	4-hydroxyphenylpyruvate dioxygenase
5	PST0265	*osmC*	0.48	Up	osmotically inducible protein OsmC
6	PST0266		0.30	Up	ribonucleotide reductase, alpha subunit
7	PST0349	*ntrC*	0.29	Up	nitrogen regulation protein NtrC
8	PST0350	*ntrB*	0.35	Up	nitrogen regulation protein NtrB
9	PST0353	*glnA*	0.33	Up	glutamine synthetase
10	PST0446		0.45	Up	cytoplasmic membrane protein
11	PST0502	*glnK*	0.13	Up	nitrogen regulatory protein P-II
12	PST0503	*amtB1*	0.24	Up	ammonium transporter
13	PST0504	*amtB2*	0.44	Up	ammonium transporter
14	PST0565		0.42	Up	major facilitator family transporter
15	PST0571		0.41	Up	conserved hypothetical protein
16	PST0585		0.48	Up	site-specific recombinase, phage integrase family
17	PST0586		0.40	Up	conserved hypothetical protein
18	PST0610		0.49	Up	dihydroxyacid dehydratase/phosphogluconate dehydratase
19	PST0630		0.46	NR	conserved hypothetical protein
20	PST0632		0.33	Up	type I restriction-modification system, S subunit
21	PST0692	*phaP*	0.39	Up	phasin PhaP
22	PST0722	*prkA*	0.49	Up	serine protein kinase PrkA
23	PST0754		0.39	Up	membrane protein
24	PST0764		0.41	NR	transcriptional activator, putative
25	PST0811	*katA*	0.44	Up	Catalase
26	PST0813		0.35	Up	major facilitator family transporter
27	PST0856		0.41	Up	conserved hypothetical protein
28	PST0874	*pctA*	0.44	Up	chemotactic transducer PctA
29	PST0937		0.45	Up	conserved hypothetical protein

30	PST0949		0.46	Up	conserved hypothetical protein
31	PST1053		0.47	NR	conserved hypothetical protein
32	PST1055	*pilB*	0.50	NR	type 4 fimbrial biogenesis protein PilB
33	PST1140		0.47	Up	conserved hypothetical protein
34	PST1172		0.30	NR	PhoH family protein
35	PST1179		0.40	NR	conserved hypothetical protein
36	PST1273		0.49	Up	putative membrane protein
37	PST1279		0.26	Up	conserved hypothetical protein
38	PST1280		0.26	NR	conserved hypothetical protein
39	PST1301	*cobS*	0.13	Up	cobalamin (5'-phosphate) synthase
40	PST1302		0.06	Up	glutaredoxin-related protein
41	PST1303		0.03	Up	thiosulfate sulfurtransferase glpE
42	PST1304	*nifQ*	0.03	Up	nitrogen fixation protein NifQ
43	PST1305		0.03	Up	arsenate reductase related protein
44	PST1306	*nifB*	0.06	Up	FeMo cofactor biosynthesis protein NifB
45	PST1308		0.43	Up	transcriptional regulator, LysR family
46	PST1312	*tpmA*	0.34	Up	thiopurine s-methyltransferase
47	PST1313	*nifA*	0.15	Up	nitrogen fixation positive regulatory protein
48	PST1314	*nifL*	0.12	Up	nitrogen fixation negative regulatory protein
49	PST1315	*rnfA*	0.37	Up	electron transport complex, RnfABCDGE type, A subunit
50	PST1316	*rnfB*	0.10	Up	electron transport complex, RnfABCDGE type, B subunit
51	PST1317	*rnfC*	0.49	Up	electron transport complex, RnfABCDGE type, C subunit
52	PST1318	*rnfD*	0.13	Up	electron transport complex, RnfABCDGE type, D subunit
53	PST1319	*rnfG*	0.16	Up	electron transport complex, RnfABCDGE type, G subunit
54	PST1320	*rnfE*	0.15	Up	electron transport complex, RnfABCDGE type, E subunit
55	PST1321	*rnfH*	0.21	Up	electron transport complex, RnfABCDGE type, H subunit
56	PST1322	*nifY2*	0.10	Up	dinitrogenase iron-molybdenum cofactor biosynthesis
57	PST1323		0.18	Up	nitrogen fixation-related protein
58	PST1324		0.15	Up	conserved hypothetical protein
59	PST1325		0.15	Up	conserved hypothetical protein
60	PST1326	*nifH*	0.32	Up	Fe protein, nitrogenase reductase NifH
61	PST1327	*nifD*	0.56	Up	MoFe protein, alpha subunit
62	PST1328	*nifK*	0.62	Up	MoFe protein, beta subunit
63	PST1330	*nifY*	0.33	Up	nitrogenase iron-molybdenum cofactor biosynthesis
64	PST1331		0.41	Up	conserved hypothetical protein
65	PST1333	*nifE*	0.05	Up	nitrogenase iron-molybdenum cofactor biosynthesis protein
66	PST1334	*nifN*	0.07	Up	nitrogenase iron-molybdenum cofactor biosynthesis protein
67	PST1335	*nifX*	0.10	Up	nitrogenase iron-molybdenum cofactor biosynthesis protein
68	PST1336		0.29	Up	protein of unknown function DUF269
69	PST1337		0.05	Up	protein of unknown function DUF683
70	PST1338		0.08	Up	ferredoxin, 4Fe-4S

71	PST1342		0.43	Up	conserved hypothetical protein
72	PST1344		0.21	Up	conserved hypothetical protein
73	PST1346	*modB*	0.48	Up	molybdate ABC transporter, permease protein
74	PST1347	*modA*	0.20	Up	molybdenum ABC transporter, periplasmic binding protein
75	PST1348		0.22	Up	putative molybdenum-binding protein
76	PST1349	*hesB*	0.07	Up	Fe-S cluster assembly protein
77	PST1350	*nifU*	0.11	Up	Fe-S cluster assembly protein NifU
78	PST1351	*nifS*	0.11	Up	nitrogenase metalloclusters biosynthesis proteinNifS
79	PST1352	*nifV*	0.05	Up	NifV protein, encodes a homocitrate synthase
80	PST1353	*cysE*	0.05	Up	serine acetyltransferase (cysE-like)
81	PST1354		0.10	Up	conserved hypothetical protein
82	PST1355	*nifW*	0.16	Up	nitrogenase stabilizing/protective protein nifW
83	PST1356	*nifZ*	0.17	Up	Fe-S cofactor synthesis protein
84	PST1357	*nifM*	0.19	Up	NifM protein, putative a peptidyl-prolyl cis/trans isomerase
85	PST1404	*fliC*	0.47	Up	flagellin type B
86	PST1450		0.48	NR	conserved hypothetical protein
87	PST1495		0.43	Up	TonB-dependent siderophore receptor
88	PST1503		0.39	Up	conserved hypothetical protein
89	PST1520		0.44	Up	conserved hypothetical protein
90	PST1521		0.33	Up	outer membrane protein
91	PST1528		0.48	NR	outer membrane lipoprotein, putative
92	PST1561		0.47	Up	ribosomal subunit interface protein, putative
93	PST1563	*adhC*	0.49	Up	alcohol dehydrogenase class III
94	PST1595		0.42	NR	ribosomal subunit interface protein, putative
95	PST1642		0.39	Up	rhodanese domain protein
96	PST1643		0.46	Up	conserved hypothetical protein
97	PST1644		0.42	Up	conserved hypothetical protein
98	PST1711	*exbD1*	0.43	Up	TonB system transport protein
99	PST1712	*exbB1*	0.35	Up	TonB system transport protein ExbB
100	PST1713		0.06	Up	TonB protein, C-terminal domain
101	PST1714		0.44	Up	predicted Zn-dependent protease
102	PST1715		0.28	Up	TldD/PmbA family protein
103	PST1887		0.50	NR	5-oxo-L-prolinase, putative
104	PST1890		0.19	NR	probable chemotaxis transducer
105	PST1920		0.29	Up	conserved hypothetical protein
106	PST1954		0.28	Up	nitrite reductase [NAD(P)H], small subunit
107	PST1955		0.05	Up	nitrite reductase [NAD(P)H] large subunit
108	PST1966		0.49	NR	conserved hypothetical protein
109	PST1992		0.31	Up	ABC transporter, periplasmic substrate-binding protein
110	PST1993		0.24	Up	glutamate--ammonia ligase
111	PST2002		0.44	Up	conserved hypothetical protein

112	PST2003		0.35	Up	ABC-type nitrate/sulfonate/bicarbonate transport systems, periplasmic component
113	PST2048		0.41	Up	universal stress protein family
114	PST2137	*glgA*	0.41	Up	glycogen synthase
115	PST2154		0.45	Up	alpha-amylase family protein
116	PST2234		0.19	Up	heat shock protein, HSP20 family
117	PST2270		0.49	NR	quinoprotein alcohol dehydrogenase
118	PST2335		0.44	Up	Aminotransferase
119	PST2381		0.28	Up	conserved hypothetical protein
120	PST2400	*nasS*	0.44	Up	nitrate-binding protein NasS
121	PST2402		0.16	Up	conserved hypothetical protein
122	PST2406	*nasA*	0.15	Up	nitrate transporter
123	PST2409		0.09	Up	assimilatory nitrite reductase large subunit
124	PST2410		0.22	Up	assimilatory nitrite reductase small subunit
125	PST2411		0.43	Up	assimilatory nitrate reductase
126	PST2425		0.41	Up	conserved hypothetical protein
127	PST2496		0.33	Up	conserved hypothetical protein
128	PST2498		0.37	Up	conserved hypothetical protein
129	PST2501		0.48	Up	capsular polysaccharide biosynthesis protein
130	PST2508		0.21	Up	methyl-accepting chemotaxis transducer
131	PST2545		0.45	NR	signal transduction histidine kinase
132	PST2747		0.34	Up	chromosome segregation ATPase
133	PST2748		0.42	Up	OmpA family protein
134	PST2769	*bfrB*	0.47	NR	Bacterioferritin
135	PST2837		0.49	Up	conserved hypothetical protein
136	PST2862		0.18	Up	nucleoside-binding outer membrane protein
137	PST2897		0.39	Up	probable oxidoreductase
138	PST2899		0.19	Up	hypothetical protein
139	PST2900		0.33	Up	probable ABC transporter, ATP-binding component
140	PST2906		0.24	Up	conserved hypothetical protein
141	PST2907		0.28	Up	ABC transporter, ATP-binding protein
142	PST2913	*topB*	0.32	Up	DNA topoisomerase III
143	PST2982	*braC*	0.46	Up	branched-chain amino acid transport protein BraC
144	PST3079		0.08	Up	membrane protein, bmp family
145	PST3080		0.32	Up	oxidoreductase, 2OG-Fe(II) oxygenase family
146	PST3099		0.23	Up	nucleoside-binding outer membrane protein
147	PST3106		0.37	Up	NAD-dependent aldehyde dehydrogenase
148	PST3129		0.04	Up	conserved hypothetical protein
149	PST3130	*iciA*	0.44	Up	chromosome initiation inhibitor
150	PST3246		0.30	Up	PqiB family protein
151	PST3253		0.40	Up	membrane protein, putative

152	PST3361	*czcA*	0.28	Up	heavy metal efflux pump CzcA
153	PST3371		0.40	Up	conserved hypothetical protein
154	PST3408		0.42	Up	permease, drug/metabolite transporter (DMT) superfamily
155	PST3414		0.40	Up	conserved hypothetical protein
156	PST3416		0.39	Up	Co/Zn/Cd efflux system component
157	PST3417		0.39	Up	predicted transcriptional regulators
158	PST3420		0.50	NR	heavy metal sensor histidine kinase
159	PST3422		0.46	Up	out membrane porin
160	PST3566	*cynS*	0.28	Up	cyanate lyase
161	PST3569	*codB*	0.47	Up	cytosine transporter
162	PST3570	*codA*	0.47	Up	cytosine deaminase
163	PST3597		0.12	Up	bacterial luciferase family protein
164	PST3598		0.17	Up	isochorismatase family protein
165	PST3621		0.41	Up	transcriptional regulator, AraC family
166	PST3680		0.32	Up	ethanolamine transporter
167	PST3720		0.09	Up	branched-chain amino acid ABC transporter, periplasmic amino acid-binding protein, putative
168	PST3726	*ureD-2*	0.26	Up	urease accessory protein UreD
169	PST3727	*ureA*	0.27	Up	urease, gamma subunit
170	PST3728	*ureB*	0.45	NR	urease, beta subunit
171	PST3736	*ureE*	0.24	Up	urease accessory protein UreE
172	PST3737	*ureF-2*	0.46	Up	urease accessory protein UreF
173	PST3767		0.42	NR	conserved hypothetical protein
174	PST3780	*rodA*	0.39	Up	rod-shape-determining protein RodA
175	PST3795		0.50	Up	ribosome-associated GTPase
176	PST3909		0.33	Up	conserved hypothetical protein
177	PST3910		0.32	Up	conserved hypothetical protein
178	PST3912		0.36	Up	hypothetical protein
179	PST3934		0.46	Up	conserved hypothetical protein
180	PST4084		0.42	Up	ABC-type amino acid transport/signal transduction systems, periplasmic component/domain
181	PST4091	*nasR*	0.38	Up	nitrate-and nitrite-responsive positive regulator
182	PST4092	*nasF*	0.06	Up	NrtA-type periplasmic nitrate transport binding protein, probable
183	PST4093	*nasE*	0.24	Up	nitrate ABC transporter permease protein
184	PST4094	*nasD*	0.05	Up	nitrate ABC transporter, ATP-binding protein, putative
185	PST4095		0.21	Up	putative acetyltransferase
186	PST4124		0.42	Up	periplasmic binding protein, putative

a, relative increase in nitrogen fixation condition compared to the stationary nitrogen-excess condition. Up, means genes are up-regulated in nitrogen fixation condition with the factors ≥ 2.0; Down, genes are down-regulated in nitrogen fixation condition with the factors ≤0.5; and NR (not regulated), 0.5 ≥ ratio ≤ 2.0.
