# Supplementary material for: Global transcriptional analysis of nitrogen fixation and ammonium repression in root-associated Pseudomonas stutzeri A1501
Source: BMC Genomics. 2010 Jan 7;11:11. doi: 10.1186/1471-2164-11-11 (PMC2820453; doi:10.1186/1471-2164-11-11)
Supplement: Additional file 3 — Genes induced specifically under nitrogen fixation conditions. [file 1471-2164-11-11-S3.PDF]

Additional file 3

Genes induced specifically under nitrogen fixation conditions

Gene ID	Gene name	Functional description	RpoN consensus[a,b]	Counterparts in DJ[c]	Counterparts in BH72[c]
PST0029		conserved hypothetical protein	Yes	y	n
PST0035		coserved hypothetical protein	Yes	y	n
PST0200*		4-hydroxyphenylpyruvate dioxygenase	Yes	y	y
PST0265	*osmC*	osmotically inducible protein	Yes[b]	y	n
PST0266		ribonucleotide reductase, alpha subunit	Yes[b]	y	n
PST0349	*ntrC*	nitrogen regulation protein	Yes[b]	y	y
PST0350	*ntrB*	nitrogen regulation protein	Yes[b]	y	y
PST0353	*glnA*	glutamine synthetase	Yes	y	y
PST0446		cytoplasmic membrane protein	Yes	y	y
PST0502	*glnK*	nitrogen regulatory protein P-II	Yes[b]	y	y
PST0503	*amtB1*	ammonium transporter	Yes[b]	y	y
PST0504	*amtB2*	ammonium transporter	Yes[b]	n	y
PST0565		major facilitator family transporter	Yes	y	y
PST0571		conserved hypothetical protein	?	n	n
PST0585		site-specific recombinase	?	n	n
PST0586		conserved hypothetical protein	?	n	n
PST0610		dihydroxyacid dehydratase	Yes	n	y
PST0632		type I restriction-modification system	?	n	n
PST0692	*phaP*	phasin PhaP	?	y	y
PST0722	*prkA*	serine protein kinase PrkA	Yes	y	y
PST0754		membrane protein	Yes	y	y
PST0811	*katA*	catalase	?	n	n
PST0813		major facilitator family transporter	Yes	y	y
PST0856		conserved hypothetical protein	?	n	n
PST0874	*pctA*	chemotactic transducer PctA	Yes	n	y
PST0937		conserved hypothetical protein	Yes	n	n
PST0949		conserved hypothetical protein	?	y	n
PST1140		conserved hypothetical protein	?	y	y
PST1273		putative membrane protein	Yes	y	y
PST1279		conserved hypothetical protein	?	y	n
PST1301	*cobS*	cobalamin (5'-phosphate) synthase	Yes	y	y
PST1302		glutaredoxin-related protein	Yes[b]	y	n
PST1303		thiosulfate sulfurtransferase glpE	Yes[b]	y	y
PST1304	*nifQ*	nitrogen fixation protein NifQ	Yes[b]	y	y
PST1305		arsenate reductase related protein	Yes[b]	y	y
PST1306	*nifB*	FeMo cofactor biosynthesis protein NifB	Yes[b]	y	y
PST1308		transcriptional regulator, LysR family	?	y	y
PST1312	*tpmA*	thiopurine s-methyltransferase	Yes	y	n
PST1313	*nifA*	positive regulatory protein	Yes[b]	y	y
PST1314	*nifL*	negative regulatory protein	Yes[b]	y	y

PST1315	*rnfA*	electron transport complex, A subunit	Yes[b]	y	y
PST1316	*rnfB*	electron transport complex, B subunit	Yes[b]	y	y
PST1317	*rnfC*	electron transport complex, C subunit	Yes[b]	y	y
PST1318	*rnfD*	electron transport complex, D subunit	Yes[b]	y	y
PST1319	*rnfG*	electron transport complex, G subunit	Yes[b]	y	y
PST1320	*rnfE*	electron transport complex, E subunit	Yes[b]	y	y
PST1321	*rnfH*	electron transport complex, H subunit	Yes[b]	y	y
PST1322	*nifY2*	dinitrogenase iron-molybdenum cofactor biosynthesis protein	Yes[b]	y	y
PST1323		nitrogen fixation-related protein	Yes[b]	y	y
PST1324		conserved hypothetical protein	Yes[b]	y	y
PST1325	*pnfA*	conserved hypothetical protein	Yes[b]	y	y
PST1326	*nifH*	Fe protein	Yes	y	y
PST1327	*nifD*	MoFe protein, alpha subunit	Yes[b]	y	y
PST1328	*nifK*	MoFe protein, beta subunit	Yes[b]	y	y
PST1330	*nifY*	nitrogenase iron-molybdenum cofactor biosynthesis protein	Yes	y	y
PST1331		conserved hypothetical protein	Yes[b]	y	y
PST1333	*nifE*	nitrogenase iron-molybdenum cofactor biosynthesis protein	Yes[b]	y	y
PST1334	*nifN*	nitrogenase iron-molybdenum cofactor biosynthesis protein	Yes[b]	y	y
PST1335	*nifX*	nitrogenase iron-molybdenum cofactor biosynthesis protein	Yes[b]	y	y
PST1336		protein of unknown function DUF269	Yes[b]	y	y
PST1337		protein of unknown function DUF683	Yes[b]	y	y
PST1338		ferredoxin, 4Fe-4S	Yes	y	y
PST1342		conserved hypothetical protein	Yes[b]	y	n
PST1344		conserved hypothetical protein	Yes	y	y
PST1346	*modB*	molybdate ABC transporter	Yes[b]	y	y
PST1347	*modA*	molybdenum ABC transporter	Yes[b]	y	y
PST1348		putative molybdenum-binding protein	Yes[b]	y	y
PST1349	*hesB*	Fe-S cluster assembly protein	Yes[b]	y	y
PST1350	*nifU*	Fe-S cluster assembly protein NifU	Yes[b]	y	y
PST1351	*nifS*	nitrogenase metalloclusters biosynthesis proteinNifS	Yes[b]	y	y
PST1352	*nifV*	homocitrate synthase	Yes[b]	y	y
PST1353	*cysE*	serine acetyltransferase (*cysE*-like)	Yes[b]	y	y
PST1354		conserved hypothetical protein	Yes[b]	y	y
PST1355	*nifW*	nitrogenase stabilizing/protective protein	Yes[b]	y	y
PST1356	*nifZ*	Fe-S cofactor synthesis protein	Yes[b]	y	y
PST1357	*nifM*	putative peptidyl-prolyl cis/trans isomerase	Yes[b]	y	y
PST1404	*fliC*	flagellin type B	Yes	y	y
PST1495		TonB-dependent siderophore receptor	?	y	n
PST1503		conserved hypothetical protein	Yes	y	n

PST1520		conserved hypothetical protein	Yes	y	n
PST1521		outer membrane protein	?	y	n
PST1561		ribosomal subunit interface protein	?	y	n
PST1563	*adhC*	alcohol dehydrogenase class III	?	y	y
PST1642		rhodanese domain protein	?	y	n
PST1643		conserved hypothetical protein	?	n	n
PST1644		conserved hypothetical protein	Yes	n	n
PST1711	*exbD1*	TonB system transport protein	Yes	y	y
PST1712	*exbB1*	TonB system transport protein ExbB	Yes[b]	y	y
PST1713		TonB protein, C-terminal domain	Yes[b]	y	y
PST1714		predicted Zn-dependent protease	Yes[b]	y	n
PST1715		TldD/PmbA family protein	Yes[b]	y	n
PST1920		conserved hypothetical protein	Yes	n	n
PST1954		nitrite reductase [NAD(P)H], small subunit	Yes[b]	y	y
PST1955		nitrite reductase [NAD(P)H], large subunit	Yes[b]	y	y
PST1992		ABC transporter	Yes[b]	n	y
PST1993		glutamate-ammonia ligase	Yes[b]	y	y
PST2002		conserved hypothetical protein	Yes	n	n
PST2003		ABC-type transport systems	Yes	n	n
PST2048		universal stress protein family	?	y	n
PST2137	*glgA*	glycogen synthase	Yes	y	y
PST2154		alpha-amylase family protein	Yes	y	y
PST2234		heat shock protein, HSP20 family	Yes	y	n
PST2335		aminotransferase	Yes	y	y
PST2381		conserved hypothetical protein	Yes	y	y
PST2400	*nasS*	nitrate-binding protein NasS	Yes	y	y
PST2402		conserved hypothetical protein	?	n	n
PST2406	*nasA*	nitrate transporter	Yes	y	y
PST2409		assimilatory nitrite reductase large subunit	Yes[b]	y	y
PST2410		assimilatory nitrite reductase small subunit	Yes[b]	y	n
PST2411		assimilatory nitrate reductase	Yes[b]	y	y
PST2425		conserved hypothetical protein	Yes	y	n
PST2496		conserved hypothetical protein	Yes	n	y
PST2498		conserved hypothetical protein	?	n	n
PST2501		capsular polysaccharide biosynthesis protein	Yes	y	y
PST2508		methyl-accepting chemotaxis transducer	Yes	n	n
PST2747		chromosome segregation ATPase	Yes[b]	y	n
PST2748		OmpA family protein	Yes[b]	y	y
PST2837		conserved hypothetical protein	Yes	y	n
PST2862		nucleoside-binding outer membrane protein	Yes	n	y
PST2897		probable oxidoreductase	Yes	n	n
PST2899		hypothetical protein	Yes	y	y
PST2900		probable ABC transporter	Yes	y	y
PST2906		conserved hypothetical protein	Yes	n	n

PST2907		ABC transporter, ATP-binding protein	Yes	y	y
PST2913	*topB*	DNA topoisomerase III	Yes	y	y
PST2982	*braC*	branched-chain amino acid transport protein	?	y	y
PST3079		membrane protein, bmp family	Yes	n	y
PST3080		oxidoreductase	Yes	n	n
PST3099		nucleoside-binding outer membrane protein	Yes	n	y
PST3106		NAD-dependent aldehyde dehydrogenase	Yes	n	n
PST3129		conserved hypothetical protein	Yes	n	n
PST3130	*iciA*	chromosome initiation inhibitor	Yes	n	n
PST3246		PqiB family protein	Yes	y	n
PST3253		membrane protein, putative	Yes	y	y
PST3361	*czcA*	heavy metal efflux pump CzcA	Yes	y	y
PST3371		conserved hypothetical protein	?	n	n
PST3408		permease	Yes	n	n
PST3414		conserved hypothetical protein	?	n	n
PST3416		Co/Zn/Cd efflux system component	?	n	n
PST3417		predicted transcriptional regulators	Yes	n	n
PST3422		out membrane porin	Yes	y	n
PST3566	*cynS*	cyanate lyase	Yes	n	n
PST3569	*codB*	cytosine transporter	Yes[b]	n	n
PST3570	*codA*	cytosine deaminase	Yes[b]	y	y
PST3597		bacterial luciferase family protein	Yes[b]	y	y
PST3598		isochorismatase family protein	Yes[b]	y	n
PST3621		transcriptional regulator, AraC family	?	y	y
PST3680		ethanolamine transporter	Yes	n	n
PST3720		branched-chain amino acid ABC transporter, putative	Yes	n	y
PST3726	*ureD*	urease accessory protein UreD	Yes[b]	y	y
PST3727	*ureA*	urease, gamma subunit	Yes[b]	y	y
PST3736	*ureE*	urease accessory protein UreE	Yes[b]	y	y
PST3737	*ureF*	urease accessory protein UreF	Yes[b]	y	y
PST3780	*rodA*	rod-shape-determining protein RodA	?	y	y
PST3795		ribosome-associated GTPase	Yes	y	y
PST3909		conserved hypothetical protein	Yes[b]	n	n
PST3910		conserved hypothetical protein	Yes[b]	n	n
PST3912		hypothetical protein	?	y	y
PST3934		conserved hypothetical protein	Yes	y	n
PST4084		ABC-type amino acid transport/signal transduction systems	?	n	n
PST4091	*nasR*	nitrate-and nitrite-responsive positive regulator	Yes	n	y
PST4092	*nasF*	NrtA-type periplasmic nitrate transport binding protein	Yes[b]	y	y
PST4093	*nasE*	nitrate ABC transporter permease protein	Yes[b]	y	y
PST4094	*nasD*	nitrate ABC transporter	Yes[b]	y	y
PST4095		putative acetyltransferase	Yes	y	n
PST4124		periplasmic binding protein, putative	Yes	n	y

[a]ORFs preceded by a consensus sequence that resembles the σ^{54} consensus sequence for A1501. Only sequences fully match the consensus sequence GG-N_{10}-GC and lying within 300 bp upstream of predicted ORFs were considered σ^{54}-dependent promoters. ?, genes or operons are not preceded by a σ^{54} consensus promoter that matches the criteria given above.

[b]ORFs likely to form an operon.

[c]The presence or absence of the *P. stutzeri* A1501 genes in the two other sequenced genomes was assessed based on the following criterion: y, gene present, identity between gene products ≥ 30% and the match length ≥ 60% of the full length; n, gene absent, identity between gene products ≤ 30% or the match length ≤ 60% of the full length.

*Genes shaded in grey were referred as a core subset of genes induced specifically under nitrogen fixation conditions.
